# LncRNA-MTA2TR functions as a promoter in pancreatic cancer via driving deacetylation-dependent accumulation of HIF-1α

**DOI:** 10.7150/thno.34559

**Published:** 2019-07-09

**Authors:** Zhu Zeng, Feng-yu Xu, Hai Zheng, Ping Cheng, Qing-yong Chen, Zeng Ye, Jian-xin Zhong, Shi-jiang Deng, Ming-liang Liu, Kang Huang, Qiang Li, Wei Li, Yu-hang Hu, Fan Wang, Chun-you Wang, Gang Zhao

**Affiliations:** 1 Department of Emergency Surgery, Union Hospital, Tongji Medical College, Huazhong University of Science and Technology, Wuhan 430022, China.; 2 Department of Pancreatic Surgery, Union Hospital, Tongji Medical College, Huazhong University of Science and Technology, Wuhan 430022, China.

**Keywords:** pancreatic cancer, LncRNA-MTA2TR, MTA2, ATF3, HIF-1α

## Abstract

**Rationale**: Hypoxia has been proved to contribute to aggressive phenotype of cancers, while functional and regulatory mechanism of long noncoding RNA (lncRNA) in the contribution of hypoxia on pancreatic cancer (PC) tumorigenesis is incompletely understood. The aim of this study was to uncover the regulatory and functional roles for hypoxia-induced lncRNA-MTA2TR (MTA2 transcriptional regulator RNA, AF083120.1) in the regulation of PC tumorigenesis.

**Methods**: A lncRNA microarray confirmed MTA2TR expression in tissues of PC patients. The effects of MTA2TR on proliferation and metastasis of PC cells and xenograft models were determined, and the key mechanisms by which MTA2TR promotes PC were further dissected. Furthermore, the expression and regulation of MTA2TR under hypoxic conditions in PC cells were assessed. We also assessed the correlation between MTA2TR expression and PC patient clinical outcomes.

**Results**: We found that metastasis associated protein 2 (MTA2) transcriptional regulator lncRNA (MTA2TR) was overexpressed in PC patient tissues relative to paired noncancerous tissues. Furthermore, we found that depletion of MTA2TR significantly inhibited PC cell proliferation and invasion both in vitro and in vivo. We further demonstrated that MTA2TR transcriptionally upregulates MTA2 expression by recruiting activating transcription factor 3 (ATF3) to the promoter area of MTA2. Consequentially, MTA2 can stabilize the HIF-1α protein via deacetylation, which further activates HIF-1α transcriptional activity. Interestingly, our results revealed that MTA2TR is transcriptionally regulated by HIF-1α under hypoxic conditions. Our clinical samples further indicated that the overexpression of MTA2TR was correlated with MTA2 upregulation, as well as with reduced overall survival (OS) in PC patients.

**Conclusions**: These results suggest that feedback between MTA2TR and HIF-1α may play a key role in regulating PC tumorigenesis, thus potentially highlighting novel avenues PC treatment.

## Introduction

Pancreatic adenocarcinoma is among the deadliest and most aggressive cancers, with a very low 5-year survival of less than 7% 1. While there have been many efforts to develop novel PC therapeutics, overall patient survival has not improved in recent decades 2. Although more traditional efforts based on modulating tumorigenic processes at the genetic level have made, recent advances have yielded a wealth of epigenetic insights suggesting that many mechanisms including DNA methylation and long non-coding RNAs (lncRNAs) may be key regulators of oncogenesis and may thus represent novel and untapped therapeutic strategies for treating PC patients and improving clinical outcomes 3-5.

Many recent studies have identified key roles for lncRNAs in essential biological processes such as cellular proliferation, differentiation, and maintenance of pluripotency in stem cells all of which can be essential to effective tumor progression. Indeed, there have been several studies to date revealed that lncRNAs are essential for the progression of certain cancers, acting as epigenetic regulators at transcriptional or posttranscriptional levels 6-8. A large number of studies have shown that lncRNAs can serve as diagnostic and prognostic biomarkers, and may represent an attractive therapeutic target in pancreatic tumors, and especially in PC 9. Specifically, numerous lncRNAs were involved in progression of PC. As an example, lncRNA-HOTAIR is highly upregulated and promotes cellular proliferation and invasion, further disrupting apoptosis and thus serving an oncogenic function in PC cells 10-12. LncRNA-LOC389641 promotes PC progression and metastasis via regulating E-cadherin with the involvement of TNFRSF10A 13. Elevated levels of lncRNA-MEG3 suppress PC by regulating PI3K signaling pathways 14. LncRNA-XIST promotes PC proliferation through functioning as competitive endogenous RNA (ceRNA) to relieve the inhibition of miR-133a on EGFR 15. These studies indicate that lncRNAs play a wide range of functions in mediating PC initiation and progression. Moreover, other researchers have found that lncRNA polymorphisms are also associated with the incidence of PC 16. Nevertheless, the regulatory mechanism and functions of lncRNAs in PC need further investigation to supply potential therapeutic targets.

In this study, we had identified a series lncRNAs that was aberrantly expressed in PC and paired paracancerous tissues through lncRNA microarray analysis. Specifically, we focus on an upregulated lncRNA-MTA2TR, which is approximate to metastasis-associated protein 2 (MTA2). We further reveal that MTA2TR promotes PC cell proliferation and invasion by regulating MTA2 expression. Moreover, the RIP and RNA-pulldown experiments validated an interaction between MTA2TR and activating transcription factor 3 (ATF3) which activates MTA2 transcription. Meanwhile, we also reveal MTA2TR enhances the stability of HIF-1α protein via MTA2-induced deacetylation. Furthermore, we demonstrate that MTA2TR is transcriptionally upregulated by HIF-1α under hypoxic conditions. Therefore, our study implicates a reciprocal regulation of HIF-1α and MTA2TR contributing to PC progression.

## Methods

### Cell culture

The PANC-1, BxPC-3 and SW1990 cell lines were acquired from American Type Culture Collection. DNA fingerprinting was used to confirm accurate cell identity, and cells were grown for no more than 6 months after thawing. RPMI media containing 10% FBS and penicillin/streptomycin was used for all cell culture. Standard growth was conducted in a 5% CO_2_ 37 °C environment. Hypoxic treatment was carried out in 1% O_2_, 5% CO_2_, and 94% N_2_ at 37 °C. If no special instructions, treatment time is 24 h.

### Clinical samples

We obtained 40 cases of matched cancer and paracancerous tissue samples from PC patients in Union Hospital (Wuhan, China) who were either receiving a pancreatectomy or those undergoing other palliative surgical operations such as the implantation of I^125^ seeds, choledochojejunostomy, or gastroenterostomy. The control samples were obtained from the paracancerous tissues of resection or palliative surgery patients with a tissue biopsy gun (Bard Peripheral Vascular Inc). Patients met with the National Comprehensive Cancer Network 2012 guideline for PC. Histopathology was used to confirm this diagnosis. Collected tissue samples were all either snap frozen in liquid nitrogen or embedded in paraffin. All patients provided written informed consent, and this study was overseen and approved by the Ethics Committee of the Academic Medical Center of Huazhong University of Science and Technology (Permission No: 2013, S199).

### Microarray

Total RNA was isolated with Trizol from tissues. Then the RNA samples were reliably amplified and labeled to generate cDNA with the Quick Amp Labeling Kit, One-Color (Agilent). A Low Input Quick-Amp Labeling Kit (Agilent) was then used to clean and label the cDNA samples. Samples were further purified and then served as probes for microarray hybridization at 65 °C for 17 h via the Gene Expression Hybridization Kit (Agilent) with hybridization chamber gasket slides (Agilent). After washing, a microarray scanner (Agilent) was then used to scan all microarrays.

Co-expressed functional modules were identified via the Markov cluster algorithm (MCL). Values presented are log2 RMA signal intensity. The lncRNAs with log2 changing fold > 2 and p<0.05 were regarded as differentially expressed lncRNAs. Cluster 3.0 was used to produce heat maps of differentially regulated genes.

### Chromatin immunoprecipitation (ChIP)

ChIP was conducted using the EZ-ChIPTM Chromatin Immunoprecipitation Kit (Millipore, USA) based on provided directions. Corresponding Rabbit-IgG (Cell Signaling Technology, USA) served as controls. Bound DNA fragments were amplified by PCR reactions, and then analyzed via 2% agarose gel electrophoresis. Primer sequences are given in Table S4.

### RNA-Fluorescence in situ hybridization (RNA-FISH)

The FISH Tag™ RNA Multicolor Kit (Invitrogen) and MAXIscript® Kit (Ambion) were used for single molecule RNA FISH based on provided directions 17, 18. A red fluorescent probe was used to identify MTA2TR. Formaldehyde and Triton X-100 were used to fix cells. Hybridization with the probes was conducted overnight at 55 °C. Probe sequences are given in Table S4.

### RNA pull-down

The Pierce™ Magnetic RNA-Protein Pull-Down Kit (Thermo Scientific, USA) and MAXIscript® Kit (Ambion, USA) were used for RNA pull-down based on provided directions 19, 20. Primer sequences are given in Table S4.

T7 phage RNA polymerases were employed for in vitro transcription using DNA templates mixed together with RNA polymerase, rNTPs, and transcription buffer. Transcription reactions were allowed to proceed for up to 1 h at 37 °C.

RNA pull-down assay, biotin-labeled AR 3'UTR, MTA2TR-RNAs and MTA2TR-AS-RNAs were captured for 30 min at room temperature with streptavidin magnetic beads (50 µL) in RNA Capture Buffer. Beads were nixed with 40 µg of BxPC-3 and SW1990 extracts for 45 min at 4 °C. Wash buffer was then used for three rounds of washing, followed by elution at 37 °C for 15 min using Biotin Elution Buffer. Then the retrieved proteins were detected by Western blot.

### RNA immunoprecipitation (RIP)

RIP assay was conducted with Magna RIP™ RNA-Binding Protein Immunoprecipitation Kit (Millipore, USA) based on provided direction 21, 22. Cell lysis was performed in the presence of protease and RNase inhibitors. Magnetic beads were then pre-incubated with IgG, SNRNP70 or anti-ATF3 antibody (Abcam, ab207434) separately for 30 min at room temperature. The prepared cell lysates then underwent bead-bound antibody-mediated immunoprecipitation at 4 °C overnight. Then immobilize magnetic beads bound antibody-protein complexes were obtained, washed off unbound materials, RNA was purified from these complexes, and then was analyzed by qRT-PCR. Primer sequences are given in Table S4.

### Western blot and co-immunoprecipitation

Protein extracts were denatured, underwent 10% SDS-PAGE gel electrophoresis, and were then transferred to PVDF membranes (Millipore) and probed with the following primary antibodies: MTA2 (Proteintech, 66195-1-Ig), ATF3 (Abcam, ab207434), HIF-1α (Proteintech, 20960-1-AP), PAN-AC (Abconal, A2391), GAPDH (Proteintech, 10494-1-AP), β-actin (Proteintech, 20536-1-AP).

For Co-immunoprecipitation, lysates were mixed with control mouse/rabbit IgG or primary antibodies at 4 °C overnight, after which they were combined with Protein A/G PLUS-Agarose (Santa Cruz Biotechnology, sc2003) at 4 °C for 2 h. After this time, the agarose in these samples was isolated and washed with lysis buffer, and then equal sample volumes were assessed by Western blot as above.

### Fluorescence immunocytochemical staining

4% paraformaldehyde was used in order to fix cells grown on coverslips for 15 min. The fixed cells were then blocked for 30 min using 10% FBS, after which they were probed using primary antibodies for 2 h, followed by secondary FITC-labelled anti-mouse Ig or Cy3-labeled anti-rabbit Ig (Jackson Immuno Research) for 50 min. 4', 6-diamidino-2-phenylindole (DAPI; Sigma) was used to stain the nuclear DNA. Mounted coverslips were assessed by Zeiss LSM510 microscopy.

### Luciferase reporter assay

MTA2TR and MTA2 promoter region (2kb sequence upstream of the transcription initiation site) was inserted into pGL3-based vectors (termed the pGL3-MTA2TR-promoter and pGL3-MTA2-promoter). A dual luciferase reporter system (Promega, USA) was used to assess luciferase activity, which was normalized to Renilla luciferase. There were 5 replicates per group, and the experiment was conducted independently three times.

### Xenograft assay

Transfected LV-siMTA2TR#1, LV-siMTA2TR#2, or LV-siNC BxPC-3/SW1990 (2×10^6^ cells/mouse) cells were subcutaneously implanted in the right flank of nude BALB/c mice (4 weeks old; HFK Bio-Technology Co., Ltd, Beijing, China). There were a total of five mice per group, and tumor were monitored every fourth day. 28 days after implantation, mice were sacrificed. Solid tumor tissues were removed and weighed. For metastasis model, nude mice were injected LV-siMTA2TR#1, LV-siMTA2TR#2, or LV-siNC BxPC-3/SW1990 (1×10^6^ cells/mouse) cells from the tail vein. Each group has 5 mice. 4 weeks later, tumor colony in lung and liver was quantified after HE staining and nodule was observed by histology examination.

The Animal Research Committee of the Academic Medical Center at Huazhong University of Science and Technology approved all aspects of this study. The Institutional and Animal Care and Use Committees guidelines were observed to ensure appropriate animal treatment (Permission No: 655).

### Northern Blot

We purchased a kit, DIG RNA Labeling Kit (Roche, Germany) to perform northern blot analysis for MTA2TR. Firstly, preparing MTA2TR specific DNA template containing T7 promoter sequences from RT-PCR, DNA template was purified. Then DIG-labeled the RNA probes were produced according to the kit instructions with the DNA template. DIG-labeled probes were used for hybridization to nylon membrane blotted total RNA, after which they were detected with anti-digoxigenin-AP, and CSPD was used for visualization. The signals were also captured by ChemiDocTm XRS Molecular Imager system (Bio-Rad, USA).

### In Vivo Fluorescence Imaging

We used an In Vivo FX PRO system (Bruker, Germany) in order to conduct the real time fluorescent imaging of tumor-bearing mice prepared as above. After anesthetizing mice with chloral hydrate, organ fluorescence was assessed with a 465 nm excitation and a 510 nm emission wavelength. All images were exposed for 1 second.

### Statistical analysis

SPSS v13.0 was used for all analyses, and data are means ± SD. T tests were used to compare groups, while a paired T test was used to compare MTA2TR levels in paired tissue samples. Pearson's correlation analysis was used to assess the relationship between MTA2TR and MTA2 in PC tissues. *P<0.05, **P<0.01, and ***P<0.001.

### Supplementary Methods

Cell proliferation assay, Plate clone formation assay, Invasion assay, RT-PCR and qRT-PCR and Transfection assay protocols are available in the supplementary materials.

## Results

### MTA2TR overexpression correlates with PC progression

**Figure 1:**
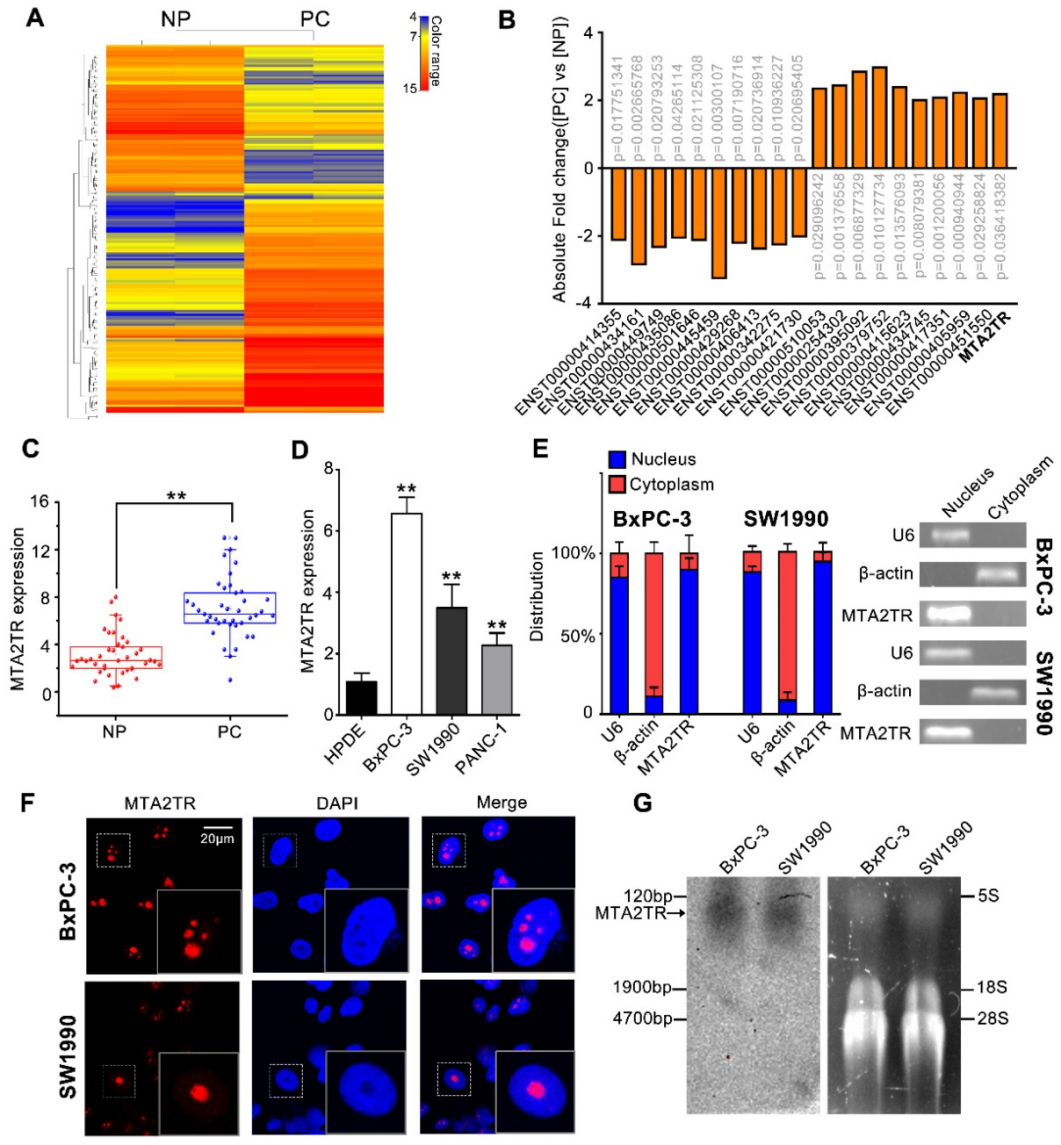
** LncRNA-MTA2TR expression is elevated in PC tissues and associated with PC progression. (A)** Microarray analysis for lncRNAs was performed with RNA extracted from PC and NP tissues. The different color represents the normalized fluorescence intensity of each lncRNA in microarray assay. **(B)** Expression change of ten upregulation lncRNAs and ten downregulation lncRNAs were validated in PC and NP tissues by Microarray analysis. **(C)** MTA2TR expression was assessed by qRT-PCR in 40 PC and paired NP tissues. The horizontal line indicates median value. **(D)** qRT-PCR analysis MTA2TR expression in the BxPC-3, SW1990, and PANC-1 PC cell lines compared with a HPDE normal human pancreatic cell line. **(E)** Subcellular distribution of MTA2TR in BxPC-3/SW1990 cells, with U6 and β-actin indicating nucleus and cytoplasm, respectively. **(F)** Single molecule RNA-FISH detection of MTA2TR (red) in BxPC-3/SW1990 cells. Nucleus was counterstained using DAPI. Scale bars: 20μm. **(G)** Northern blot with a 158-bp specific probe indicating the existence of MTA2TR transcript in BxPC-3/SW1990 cells.

To identify drivers of pancreatic tumorigenesis, differentially expressed lncRNAs in two PC samples and paired non-tumor samples were evaluated by microarray assay. The lncRNAs with log_2_ fold change >2 and p<0.05 were regarded as differentially expressed lncRNAs. Hierarchical clustering confirmed marked differences between the PC samples and the paired non-tumor peritumoral (NP) controls (Figure 1A). Among those significantly increased lncRNAs, we specifically focused on the MTA2TR (Figure 1B, Table S1). MTA2TR expression was further confirmed in 40-paired PC and NP tissues, revealing it to be more highly expressed in PC relative to NP samples (Figure 1C). Furthermore, we then compared MTA2TR expression in the BxPC-3, SW1990, and PANC-1 pancreatic cancer cell lines relative to control human pancreatic duct epithelial cell line (HPDE) cells. Overexpressed levels of MTA2TR expression were seen in all PC cell lines (Figure 1D). MTA2TR was primarily nuclear in BxPC-3 as well as in SW1990 cells (Figure 1E), which was further validated by using RNA-FISH assay (Figure 1F). In addition, the northern blot validated MTA2TR levels in the BxPC-3/SW1990 cells (Figure 1G). Next, the MTA2TR coding potential was assessed by searching Coding Potential Assessing Tool 23, which showed that the coding potential score of MTA2TR was 0.0018. From Coding Potential Calculator 24, we knew that coding probability of MTA2TR was -0.442783, further supporting that MTA2TR lacks protein-coding potential (Table S2).

### MTA2TR promotes proliferation and invasion of PC cells

**Figure 2:**
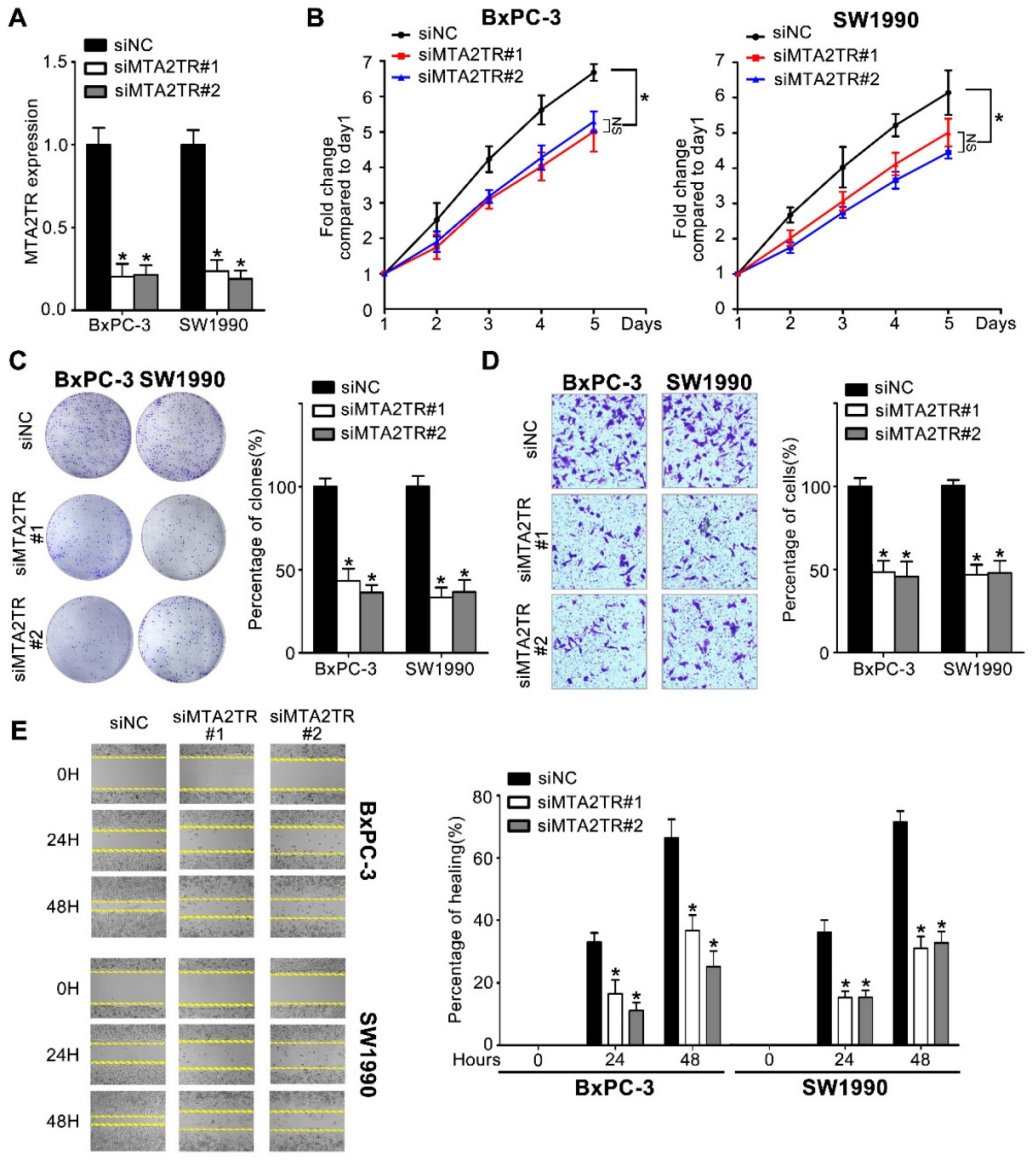
** Knockdown of MTA2TR reduced PC cell invasion and proliferation. (A)** MTA2TR knockdown efficiency in BxPC-3 cells was analyzed by qRT-PCR. **(B, C)** The effect of MTA2TR knockdown on BxPC-3 cells proliferations was measured via MTT and colony formation assay. The right histogram represents relative cell number while the representative images are shown on the left panel. Five microscopic fields were chosen at random and averaged. **(D, E)** The effect of MTA2TR knockdown on invasion and migration was analyzed respectively using Transwell and wound healing assays in BxPC-3 cells respectively. The right histogram represents relative cell number while the representative images are shown on the left panel. Five microscopic fields were chosen at random and averaged.

Since MTA2TR is overexpressed in PC cells and tissues, we used 2 kinds of siRNAs to knockdown endogenous MTA2TR expression to evaluate its roles in BxPC-3 cells as well as in SW1990 cells (Figure 2A). MTA2TR knockdown significantly disrupted BxPC-3/SW1990 cells cell proliferation and colony formation as determined via MTT and colony formation assay (Figure 2B, C). Similarly, invasion and migration of these cells were disrupted upon MTA2TR knockdown as determined based on Transwell and wound healing analyses (Figure 2D, E).

**Figure 3:**
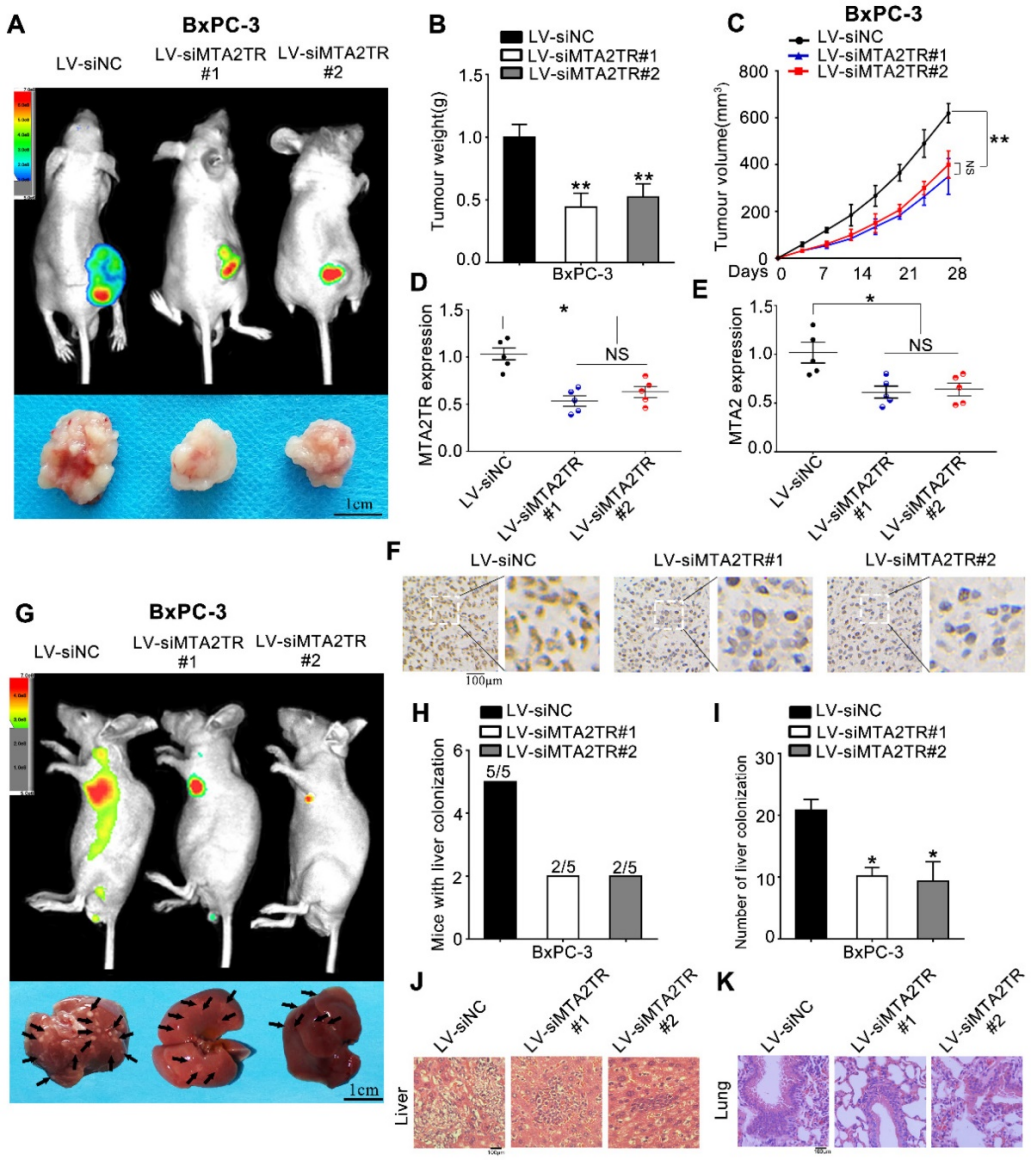
** MTA2TR facilitates the in vivo invasion and proliferation of BxPC-3 cells. (A-F)** BxPC-3 cells transfected using lentiviruses encoding siMTA2TR#1 (LV-siMTA2TR#1), siMTA2TR#2 (LV-siMTA2TR#2) or siNC (LV-siNC) were transplanted subcutaneously into nude mice. **(A)** In vivo fluorescence imaging demonstrated the subcutaneous tumor. **(B)** The weight of subcutaneous tumor was measured after 4 weeks when mice were sacrificed. **(C)** The volume of subcutaneous tumor was measured every 4 days. **(D)** MTA2TR expression was compared in the subcutaneous tumor form LV-siMTA2TR#1, LV-siMTA2TR#2 or LV-siNC group. **(E)** MTA2 mRNA expression was detected in the subcutaneous tumor form LV-siMTA2TR#1, LV-siMTA2TR#2 or LV-siNC group. **(F)** The MTA2 protein expression was demonstrated by IHC in the subcutaneous tumor form LV-siMTA2TR#1, LV-siMTA2TR#2 or LV-siNC group. **(G-K)** BxPC-3 cells transfected with LV-siMTA2TR#1, LV-siMTA2TR#2 or LV-siNC were transplanted via tail vein injection to observe tumor metastasis. **(G)** In vivo fluorescence imaging indicated the metastasis of BxPC-3 cells in LV-siMTA2TR#1, LV-siMTA2TR#2 or LV-siNC group when mice were sacrificed after 4 weeks. Arrows indicate the invasion nodules. Scale bars, 1 cm. **(H)** Liver metastasis was measured with the indicated BxPC-3 cells. N=5 mice in each group.** (I)** The number of visible liver metastases per 5 sections in each nude mouse. **(J, K)** H&E images of liver and lung tissue isolated from LV-siMTA2TR#1, LV-siMTA2TR#2 or LV-siNC group. Arrows indicate the invasion nodules. Scale bars, 100 μm.

As a means of further evaluating the biological function of MTA2TR in vivo, BxPC-3 cells were transfected stably using a lentivirus encoding either a negative control (LV-siNC), siMTA2TR#1 (LV-siMTA2TR#1) or siMTA2TR#2 (LV-siMTA2TR#2) sequences. These transfected cells were then used in order to construct a xenograft tumor model in nude mice. Compared to LV-siNC, the LV-siMTA2TR tumor growth was clearly delayed (Figure 3A-C). Importantly, the expression of MTA2TR and MTA2 in the grafted tumors were significantly downregulated in the LV-siMTA2TR group, as identified by IHC using an anti-MTA2 antibody (Figure 3D-F). We next injected nude mice with transfected BxPC-3 cells through the tail vein in order to evaluate the metastatic potential of these cells (Figure 3G). We found that both the number of mice with lung or liver metastases and the number of visible metastatic loci in the LV-siMTA2TR group were decreased relative to the control mice (Figure 3H, I), which was further confirmed by H&E staining (Figure 3J, K). We also confirmed these in vivo findings using the SW1990 cell line (Figure S1). Our results showed that siMTA2TR significantly inhibited the proliferation and metastasis of SW1990 cells in vivo which further validated the oncogenic function of MTA2TR in PC cells.

Meanwhile, we induced ectopic overexpression of MTA2TR by transfection with MTA2TR-containg plasmid in both of these PC lines (Figure S2A). After overexpression of MTA2TR, the proliferation, colony formation, transwell and wound healing assays were markedly enhanced (Figure S2B-E). Taken together, these results show that MTA2TR might enhance the proliferation and invasion of PC cells.

### MTA2TR functions through regulating MTA2 transcription

**Figure 4:**
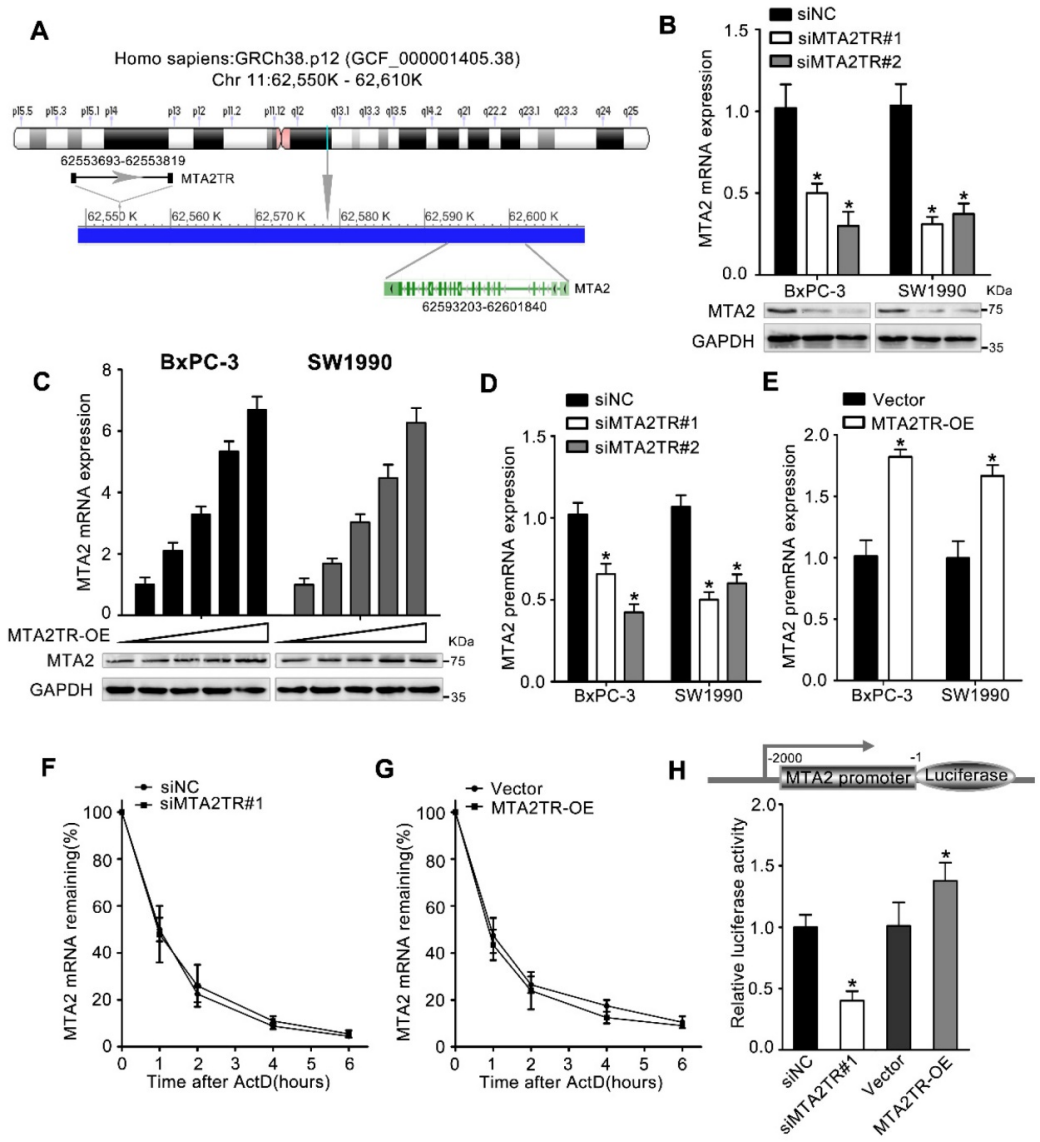
** MTA2TR predominantly functions via regulating MTA2. (A)** Schematic annotation the MTA2TR and MTA2 genomic locus. Arrows indicate transcript orientation.** (B)** Levels of MTA2 mRNA and protein in MTA2TR-knockdown BxPC-3/SW1990 cells were detected via qRT-PCR and Western blot, respectively.** (C)** BxPC-3 and SW1990 cells were transfected using empty vector as negative control (Vector) or 1.6, 3.2, 6.4, or 12.8μg MTA2TR overexpression vector (MTA2TR-OE). MTA2 mRNA and protein levels were assessed via qRT-PCR and Western blot, respectively. **(D, E)** The regulation of MTA2TR knockdown or overexpression on MTA2 premRNA expression was detected respectively. **(F, G)** Total cellular RNA was collected 0, 1, 2, 4, and 6 hours following actinomycin D treatment in MTA2TR knockdown or overexpression BxPC-3 cells and subjected to qRT-PCR. **(H)** The effect of MTA2TR-knockdown or MTA2TR-overexpression on promoter activity of MTA2 was compared via luciferase reporter assay in BxPC-3 cells co-transfected using plasmids containing wild-type MTA2 promoter.

We next wanted to determine which MTA2TR targets were resulting in the observed PC cell phenotypes. lncRNAs are well known to be able to mediate regulation of gene expression both near their own transcriptional site (in-cis) and at distinct independent loci (in-trans) 25. Since MTA2TR is adjacent to MTA2 gene, which is an oncogenic driver in numerous cancers, we wonder whether MTA2TR exerts cis-acting effect on MTA2 expression (Figure 4A). Coincidently, we revealed that MTA2TR knockdown decreased, while MTA2TR overexpression increased both mRNA and protein levels of MTA2 in PC cells (Figure 4B, C). Meanwhile, MTA2 pre-mRNA was also decreased by MTA2TR knockdown but increased after MTA2TR overexpression (Figure 4D, E). Meanwhile, stability of MTA2 mRNA had no significant changes in MTA2TR knockdown and overexpression BxPC-3 cells, which indicated MTA2 is regulated by MTA2TR but not in post-transcriptional level (Figure 4F, G). To further verify MTA2TR regulate MTA2 transcription, BxPC-3 cells were transfected using a MTA2 promoter-containing luciferase reporter vector. MTA2TR overexpression was associated with a marked increase in activity at the MTA2 promoter, while knockdown of MTA2TR impaired this promoter activity (Figure 4H). These results thus indicated that MTA2TR regulates MTA2 transcription.

Similar to what we had revealed before 26, PC cell proliferation and invasion was significantly inhibited by siMTA2, but enhanced by MTA2 overexpression obviously. Therefore, we next determine whether MTA2TR predominantly functions through its association with MTA2. Results showed that MTA2 overexpression markedly rescued the expression of MTA2, which was inhibited by MTA2TR knockdown (Figure S3A). Moreover, overexpression of MTA2 significantly rescued the proliferation and invasive ability in those MTA2TR-knockdown PC cells (Figure S3B, C). Meanwhile, MTA2 knockdown markedly reversed the expression of MTA2 in MTA2TR overexpression PC cells (Figure S4A). Moreover, MTA2 knockdown significantly reversed the proliferation and invasive ability in those MTA2TR overexpression PC cells (Figure S4B, C). Altogether, these findings suggest that MTA2 is essential for MTA2TR exerting its functions in PC cells.

### MTA2TR actives transcription of MTA2 by recruiting ATF3 to the MTA2 promoter

**Figure 5:**
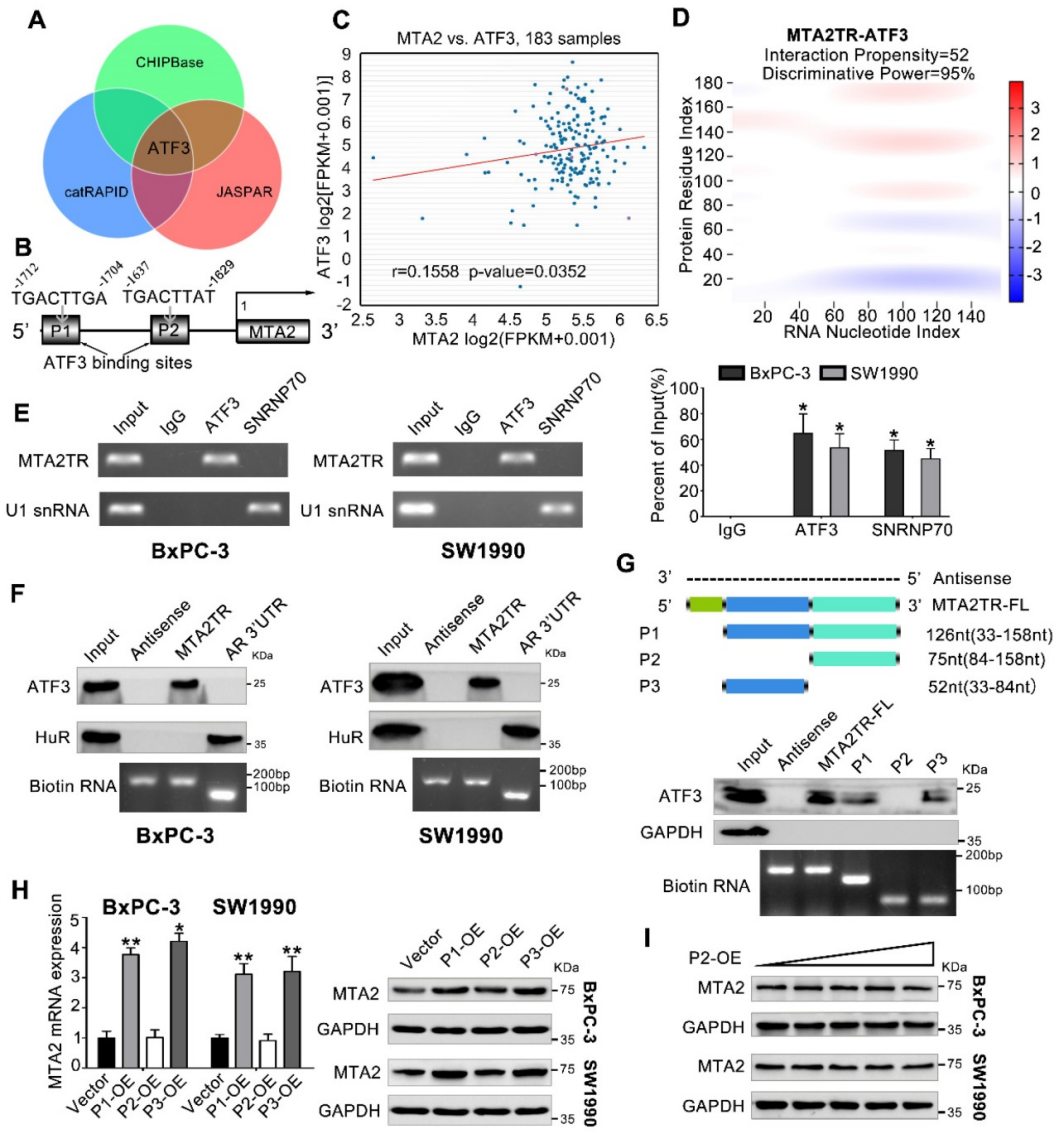
** MTA2TR is associated with transcription factor ATF3. (A)** Venn diagram of the overlap indicated ATF3 as a MTA2TR/MTA2-associated protein from ChIPBase, JASPAR, and catRAPID. **(B)** Schematic showing ATF3 binding sites (P1, P2) on the MTA2 promoter. **(C)** The correlation between ATF3 and MTA2 expression was assessed in 183 PC samples from ChIPBase.** (D)** Schematic illustration of association between MTA2TR and ATF3. Red coloration indicates stronger confidence for a given base. **(E)** The binding between MTA2TR and ATF3 was verified by RIP assay with anti-ATF3 antibody in BxPC-3/SW1990 cells, and the co-precipitated transcript were determined using qRT-PCR. Positive Control Antibody, the Anti-SNRNP70 rabbit polyclonal antibody, is expected by sequence similarity of the immunogen to cross react with SNRNP70 of human, mouse, rat and canine origins. The U1 snRNA RIP primers should also be able to amplify U1 snRNA converted cDNA in those species. **(F)** The binding between MTA2TR and ATF3 was verified by RNA-pulldown assay with a biotinylated MTA2TR probe in BxPC-3/SW1990 cells. The immunoblot analysis showed the pulldown protein with anti-ATF3 and anti-HuR antibody. RNA from the 3´ untranslated-region (UTR) of the androgen receptor (AR) served as the control, containing UC-rich HuR binding regions. MTA2TR-antisense, control RNA, lacked ATF3 or HuR binding sites. **(G)** MTA2TR deletion mutants were transcribed in vitro and applied for RNA pull-down assays. **(H)** After transfected with empty vector and plasmids containing p1, p2, p3 fragment, MTA2 mRNA and protein levels were assessed. **(I)** BxPC-3/SW1990 cells were transfected using empty vector as negative control (Vector) or 1.6, 3.2, 6.4, or 12.8μg P2-containing overexpression vector. MTA2 protein levels were measured.

We next addressed the mechanism by which MTA2TR promoted MTA2 transcription. Since MTA2TR mainly locating in nuclear, we presumed that MTA2TR might function through associating with transcriptional factor. Therefore, we performed an intersection analysis of ChIPBase, JASPAR and catRAPID database (Figure 5A). According to the JASPAR database, MTA2 promoter has two potential binding sites (P1, P2) for transcription factor ATF3 (Figure 5B). TCGA data from ChIPBase identified a positive correlation between the expression of MTA2 and ATF3 in PC (Figure 5C). Furthermore, the catRAPID database revealed an association between ATF3 and MTA2TR (Figure 5D). In addition, RNA immunoprecipitation (RIP) assay showed significant accumulation of MTA2TR transcript by anti-ATF3 antibody but not IgG and SNRNP70 (Figure 5E). Coincidently, ATF3 was detected from cell extracts of PC cells by RNA pull-down assay using MTA2TR probe (Figure 5F). The deletion-mapping analyses identified that the 33-84nt region is necessary for the binding of MTA2TR to ATF3 (Figure 5G). We also confirmed that overexpression the fragments of P1 and P3, which contains the segment (5' 33-84nt 3') of MTA2TR, but not the fragments of P2, could increase MTA2 expression both in mRNA and protein level (Figure 5H, I).

**Figure 6:**
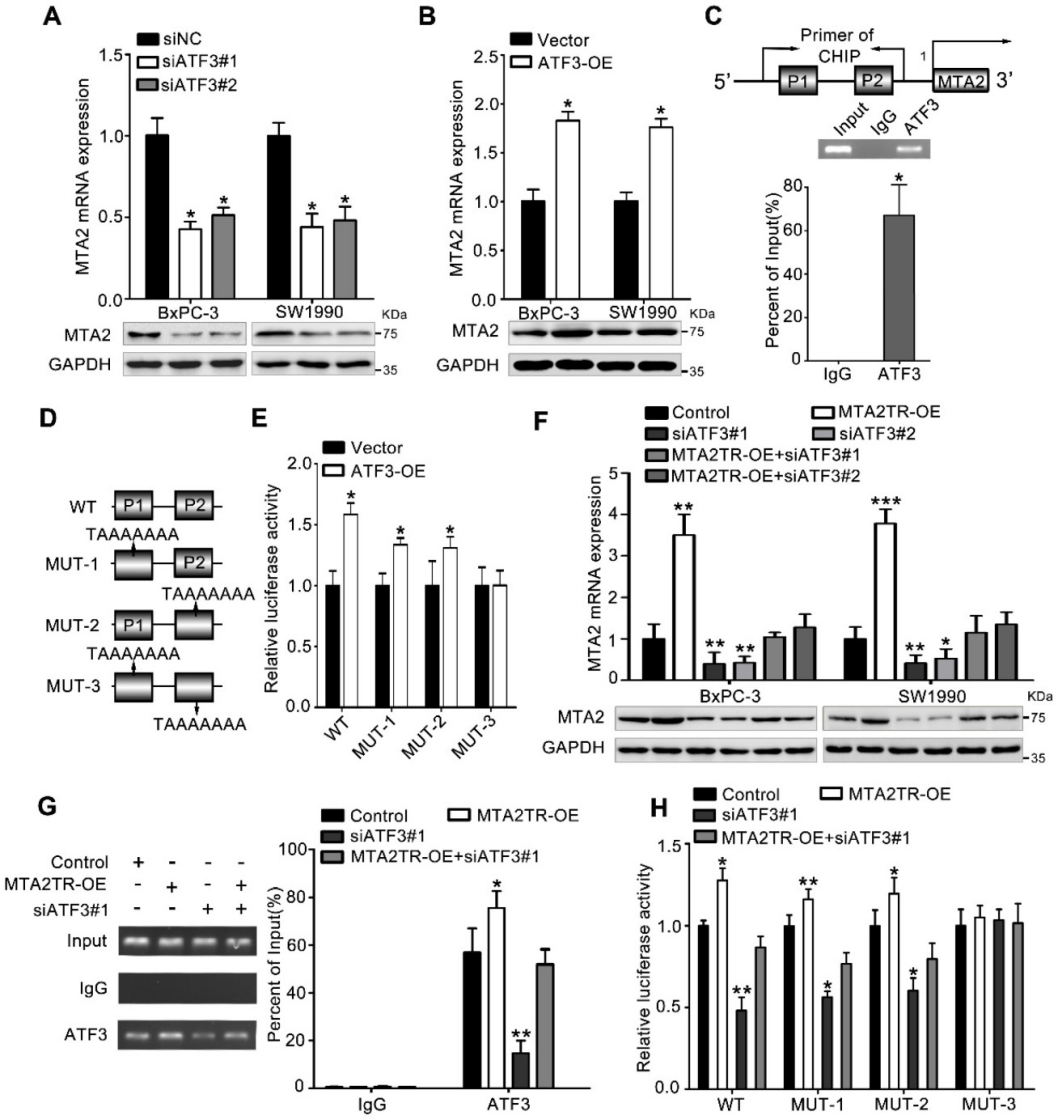
** MTA2TR regulates MTA2 transcription via recruiting ATF3 to the MTA2 promoter. (A, B)** MTA2 mRNA and protein levels were detected in ATF3-knockdown or ATF3-overexpression pancreatic cancer cells, respectively. **(C)** The binding of ATF3 on MTA2 promoter region (P1, P2) was verified with ChIP assay putative binding sites of ATF3 on the MTA2 promoter in BxPC-3 cells. **(D)** The vector containing WT and three mutants of ATF3 binding sites on the MTA2 promoter were designed for the luciferase reporter assay. **(E)** The effect of ATF3 on the MTA2 promoter activity was analyzed following transfection of BxPC-3 cells using a WT- or MUT-containing reporter vector. **(F)** MTA2 mRNA and protein levels were detected in MTA2TR-overexpression or/and ATF3-knockdown PC cells. **(G)** The binding between ATF3 and MTA2 promoter was evaluated by ChIP assay in MTA2TR-overexpression or/and ATF3-knockdown BxPC-3 cells. **(H)** The activity of MTA2 promoter was determined with a luciferase reporter assay in MTA2TR-overexpression or/and ATF3-knockdown BxPC-3 cells.

To further verify whether ATF3 is required for the regulation of MTA2TR on MTA2 transcription, ATF3 expression was modulated by a siRNA-ATF3 (siATF3) or ATF3 overexpression plasmid (ATF3-OE) (Figure S5A, B). ATF3 knockdown significantly decreased, while ATF3 overexpression increased MTA2 expression both in mRNA and protein levels (Figure 6A, B). ChIP assay showed that the MTA2 promoter segments containing both P1 and P2 were markedly accumulated by anti-ATF3 antibody but not IgG (Figure 6C). We further validated whether ATF3 activated transcription of MTA2 promoter through a luciferase reporter assay. The luciferase reporter plasmid promoter region was modified to contain wild-type (WT) or mutant binding sites (MUT1-3). Results showed the luciferase density of WT, MUT1, MUT2, but not MUT3 was markedly enhanced by ATF3 overexpression, which indicates both P1 and P2 are required for the binding between ATF3 and MTA2 promoter (Figure 6D, E). Meanwhile, the MTA2TR-induced MTA2 overexpression was dramatically inhibited by ATF3 knockdown (Figure 6F). Moreover, ATF3 knockdown reversed the binding of ATF3 on the promoter of MTA2 in MTA2TR overexpression cells (Figure 6G). Similarly, knockdown of ATF3 decreased the luciferase density in MTA2TR-overexpression cells (Figure 6H). On the contrary, ATF3 overexpression markedly rescued the binding and activating of ATF3 on MTA2 promoter, as well as the MTA2 expression, which was inhibited by MTA2TR depletion (Figure S5C-E).

In addition, overexpression of ATF3 significantly rescued the proliferation and invasive ability in those MTA2TR-knockdown PC cells (Figure S6A, B). Moreover, ATF3 knockdown significantly reversed the proliferation and invasive ability in those MTA2TR overexpression PC cells (Figure S7A, B). Therefore, these findings suggest that ATF3 is required for the regulation of MTA2TR on MTA2 transcription.

### HIF-1α induces MTA2TR overexpression during hypoxia

**Figure 7:**
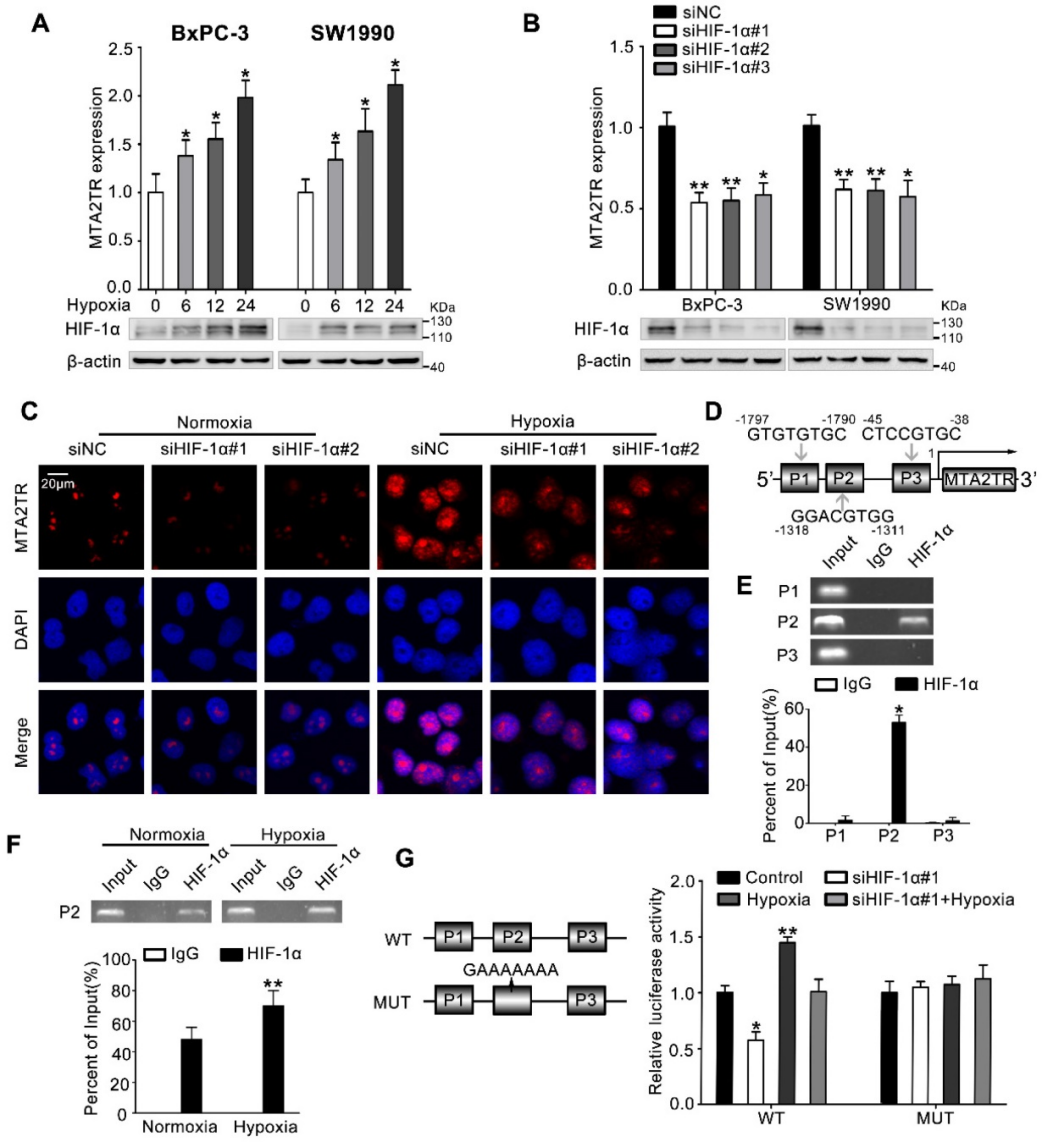
** MTA2TR is upregulated during hypoxia condition. (A)** MTA2 expression was assessed in BxPC-3/SW1990 cells during hypoxia with different time point. **(B)** MTA2TR expression was assessed in HIF-1α-knockdown BxPC-3/SW1990 cells under hypoxia condition. **(C)** The MTA2TR expression in HIF-1α-knockdown BxPC-3 cells was shown by RNA-FISH under normoxia or hypoxia condition. Nucleus was counterstained with DAPI. Scale bars: 20μm.** (D)** The putative HREs (P1, P2, and P3) on the MTA2TR promoter area was shown. **(E)** HIF-1α binding to the MTA2TR promoter was verified by ChIP assay in BxPC-3 cells. **(F)** How hypoxia affects HIF-1α binding to the MTA2TR promoter was inspected by ChIP assay in BxPC-3 cells. **(G)** Following transfection of BxPC-3 cells using a vector containing WT- and MUT-HRE of MTA2TR promoter, MTA2TR promoter activity was analyzed in the reporter cells co-transfected with siHIF-1α#1 under normoxia or hypoxia condition.

Next, we investigated the mechanism controlling MTA2TR expression in PC cells. Since we had previously demonstrated that hypoxia induces lncRNA-NUTF2P3 overexpression 27, we wonder whether the aberrant expression of MTA2TR was also attributed to the hypoxia condition of PC. MTA2TR was upregulated in PC cells upon hypoxia in a time-dependent fashion (Figure 7A). In addition, the RT-PCR results showed that HIF-1α knockdown dramatically inhibited the hypoxia-induced MTA2TR overexpression (Figure 7B), which was further validated by RNA-FISH assay (Figure 7C). Meanwhile, the Jaspar database displayed three potential hypoxia responsive elements (HREs) on the MTA2TR promoter area (Figure 7D). ChIP assay determined only the P2 region, but not P1 and P3 in the MTA2TR promoter mediates HIF-1α-binding to the MTA2TR promoter (Figure 7E). Furthermore, HIF-1α binding to the MTA2TR promoter was obviously enhanced under hypoxia (Figure 7F). To confirm functional binding of HIF-1α to the MTA2TR promoter, BxPC-3 cells were transfected with luciferase reporter vectors bearing wild-type (WT) or mutated HRE (MUT). The results showed that MTA2TR promoter activity was enhanced by hypoxia, but impaired by HIF-1α knockdown (Figure 7G). Besides, RIP assay indicated that the binding of MTA2TR and ATF3 increased under hypoxia (Figure S8A), which was further verified via a combination of ATF3 protein immunofluorescence analyses and RNA-FISH assessment of MTA2TR in BxPC-3 cells under normoxic and hypoxic conditions (Figure S8B). In addition, ChIP assay verified that the combination of ATF3 on the MTA2 promoter region was also increased (Figure S8C). Therefore, these results suggest that MTA2TR is regulated by hypoxia-induced HIF-1α at the transcriptional level.

### MTA2TR stabilizes HIF-1α via MTA2-mediated deacetylation

**Figure 8:**
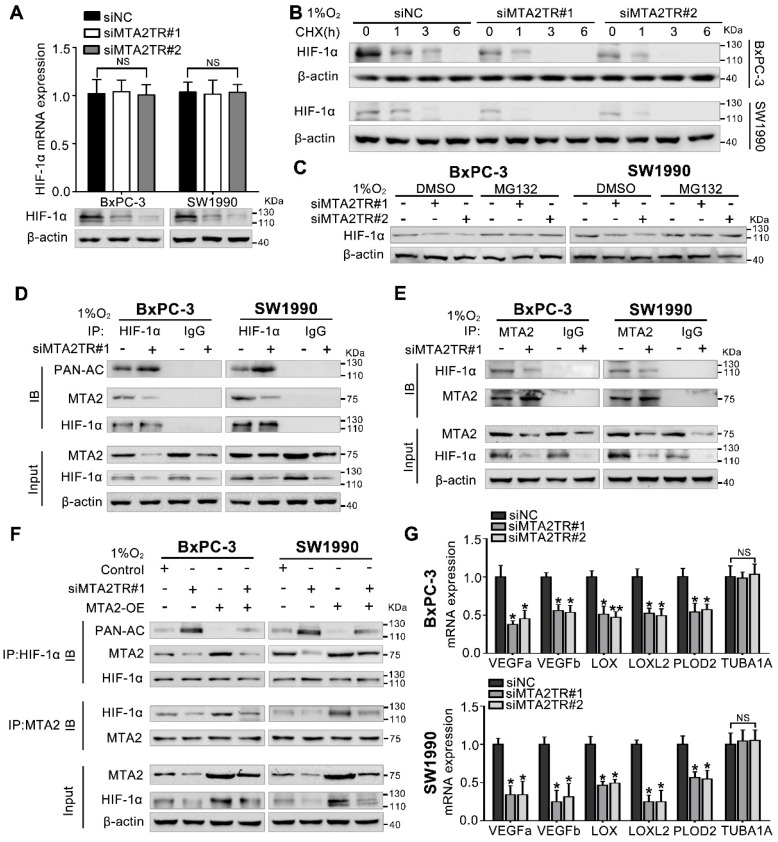
** Hypoxia-induced MTA2TR stabilizes HIF-1α by promoting deacetylation. (A)** HIF-1α mRNA and protein levels were analyzed in MTA2TR-knockdown BxPC-3/SW1990 cells.** (B)** The effect of MTA2TR knockdown on stabilization of HIF-1α was measured in BxPC-3/SW1990 cells treated with cycloheximide under hypoxia for the indicated periods of time. **(C)** HIF-1α levels in the MTA2TR-knockdown BxPC-3/SW1990 cells was detected under hypoxia with or without MG132 treatment. **(D, E)** HIF-1α acetylation and the binding between MTA2 and HIF-1α in the MTA2TR-knockdown BxPC-3/SW1990 cells treated with 1% O_2_ 24 h were analyzed in the cell lysates following anti-HIF-1α or anti-MTA2 immunoprecipitation. **(F)** The acetylation of HIF-1α and the binding between MTA2 and HIF-1α in the BxPC-3/SW1990 cells, cultured under hypoxia condition, transfected with siMTA2TR#1 or/and MTA2-OE were analyzed by IP with anti-HIF-1α or anti-MTA2 antibody. **(G)** The mRNA level of VEGFa, VEGFb, LOX, LOXL2, PLOD2 and TUBA1 was detected in MTA2TR-knockdown BxPC-3 and SW1990 cells under hypoxia condition by qRT-PCR.

Our previous study demonstrated MTA2 could promote deacetylation and stabilization of HIF-1α protein during hypoxia 26. Therefore, we further presumed whether MTA2TR can regulate HIF-1α stabilization via MTA2-dependent acetylation during hypoxia. Very interestingly, the MTA2TR knockdown significantly attenuated, while the overexpression of MTA2TR upregulated the hypoxia-induced HIF-1α protein level but without obvious effects on HIF-1α mRNA expression (Figure 8A, Figure S9A). To further reveal whether MTA2TR regulated HIF-1α stability, HIF-1α protein was assessed following cycloheximide (CHX) treatment to disrupt protein synthesis. Hypoxia-induced HIF-1α protein in PC cells was destabilized with MTA2TR knockdown, but stabilized by MTA2TR overexpression (Figure 8B, Figure S9B). Nevertheless, both MTA2TR knockdown or overexpression-induced HIF-1α modulation were abolished by the proteasome inhibitor MG132 (Figure 8C, Figure S9C). Moreover, we found that MTA2TR knockdown markedly increased acetylation of HIF-1α, accompanied with reduction of the binding of MTA2 and HIF-1α under hypoxic conditions (Figure 8D, E). On the contrary, MTA2TR overexpression decreased HIF-1α acetylation but increased association of HIF-1α and MTA2 (Figure S9D, E). Meanwhile, we verified MTA2 overexpression could rescue the MTA2TR knockdown-inhibited HIF-1α expression and association of HIF-1α and MTA2, as well as decrease the acetylation of HIF-1α (Figure 8F). On the contrary, MTA2 knockdown decreased the HIF-1α expression and association of HIF-1α and MTA2, as well as increased acetylation of HIF-1α, which were induced by MTA2TR overexpression (Figure S9F). Meanwhile, MTA2TR knockdown significantly decreased the mRNA level of HIF-1α targets including VEGFa, VEGFb, LOX, LOXL2 and PLOD2 (Figure 8G). Oppositely, mRNA levels of those HIF-1α targets were obviously upregulated by MTA2TR overexpression (Figure S9G). These results further supported that HIF-1α transcriptional activity is regulated by MTA2TR.

**Figure 9:**
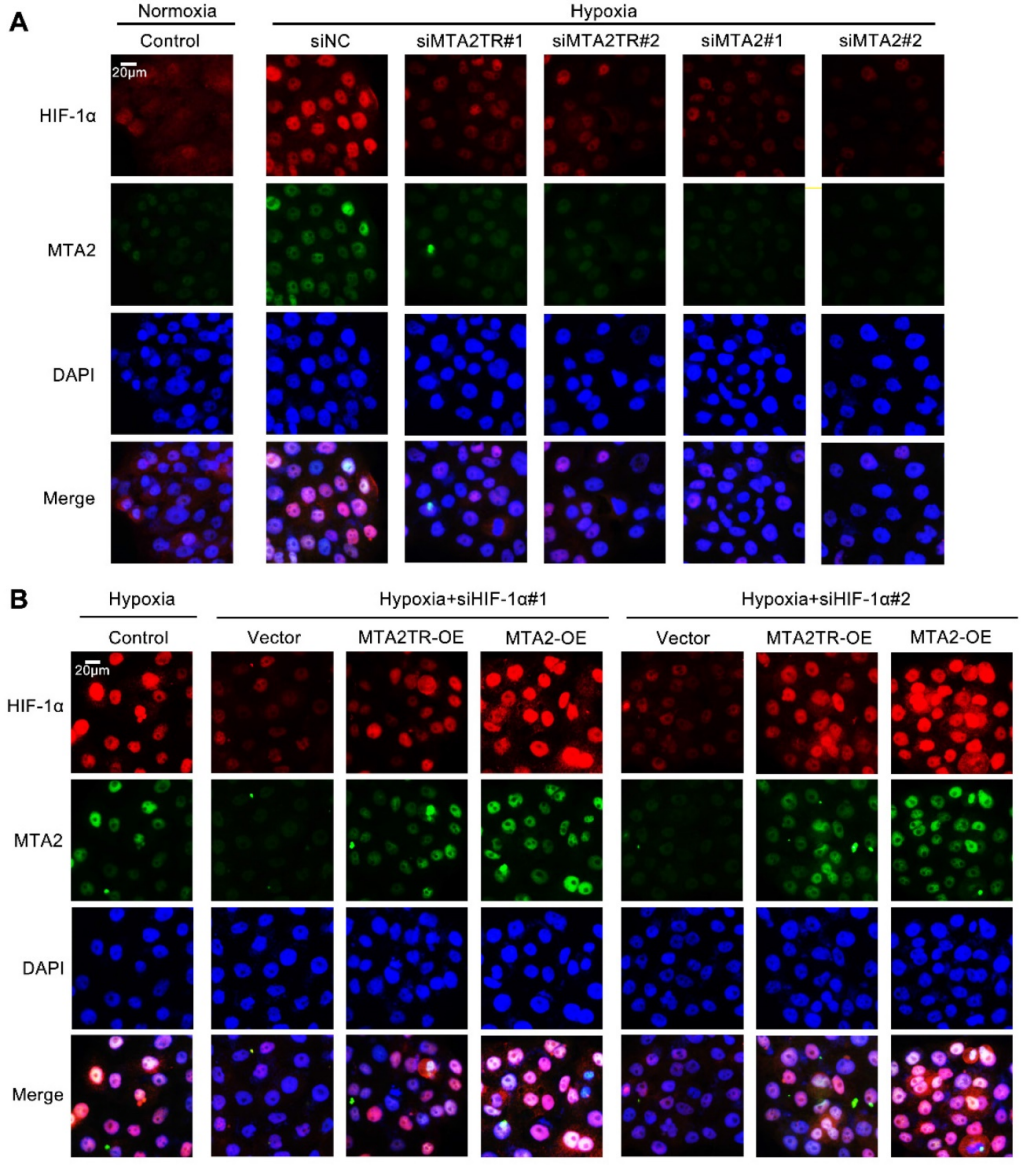
** MTA2TR is associated with the hypoxia-induced HIF-1α/MTA2 pathway. (A)** The representative immunofluorescence of MTA2 and HIF-1α in BxPC-3 cells after transfection using siMTA2TR#1, siMTA2TR#2, siMTA2#1, or siMTA2#2 under normoxia or hypoxic conditions. **(B)** The representative immunofluorescence of MTA2 and HIF-1α in BxPC-3 cells following co-transfection using siHIF-1α#1, siHIF-1α#2 and MTA2TR-OE or MTA2-OE under hypoxia conditions. Scale bars: 50 μm.

Moreover, the immunofluorescence results confirmed that the hypoxia-induced HIF-1α was decreased by knockdown of both MTA2TR and MTA2 in PC cells (Figure 9A). In addition, overexpression of MTA2TR or MTA2 could rescue the HIF-1α that was inhibited by HIF-1α knockdown during hypoxia (Figure 9B).

Then we explored the effect of MTA2TR in cell proliferation and invasion under hypoxic conditions. We assessed PC cell proliferation and invasion under hypoxic conditions using MTT and transwell experiments. Under hypoxic conditions, the cell's proliferation ability did not change. However, knockdown MTA2TR under hypoxia, the proliferation of cells is weakened (Figure S10A). We also found that hypoxia significantly increased the invasive ability of these cells (Figure S10B). Upon knockdown MTA2TR, there was a substantial decrease in the invasive potential of these PC cells (Figure S10B).

Together, these results indicate that MTA2TR increased the association between MTA2 and HIF-1α, thereafter, induced deacetylation and stabilization of HIF-1α during hypoxia.

### MTA2TR/MTA2 pathway is correlated to PC prognosis

**Figure 10:**
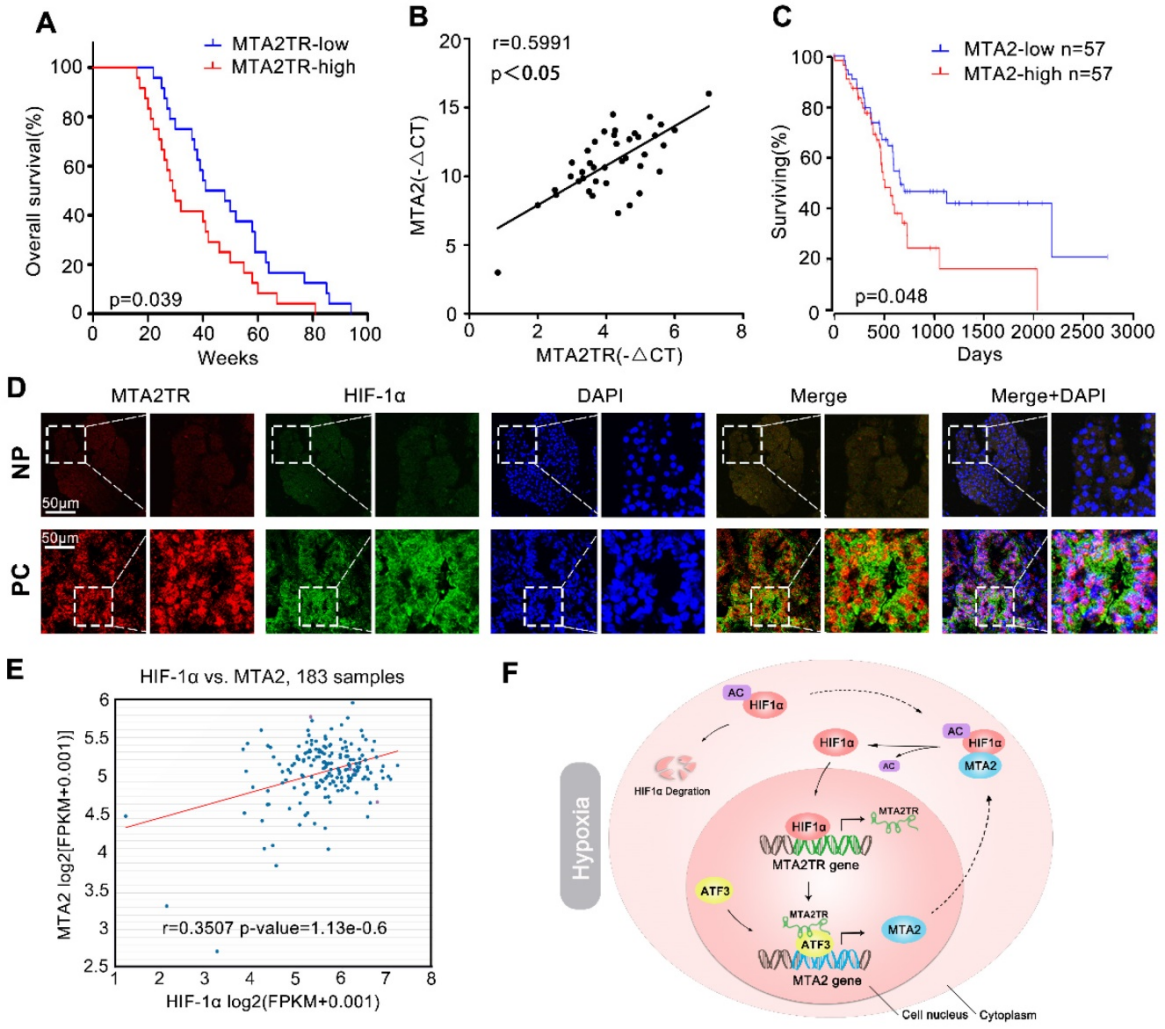
** Correlation of MTA2TR, ATF3, MTA2 and HIF-1α expression in PC samples. (A)** Kaplan-Meier curves showing overall survivals in 40 cases of PC. PC patients with MTA2TR expression level below or above the 50th percentile was classified as low or high MTA2TR group. **(B)** The Pearson's correlation between MTA2TR and MTA2 expression was analyzed in 40 PC tissues. **(C)** Kaplan-Meier curves for overall survival of 114 PC patients from OncoLnc database. MTA2 expression level below or above the 50th percentile was classified as low or high MTA2 group. **(D)** Combined immunofluorescence of HIF-1α protein (green) and RNA-FISH analysis of MTA2TR (red) in PC and corresponding NP tissues. Scale bars, 50 μm.** (E)** The correlation between HIF-1α and MTA2 expression was assessed in 183 PC samples from ChIPBase.** (F)** Graphical representation of the regulation and function of MTA2TR in PC.

We further explored the link between MTA2TR expression and clinical characteristics of PC patients. The univariate analyses revealed that overexpression of MTA2TR was correlated with tumor sizes, poor tumor differentiation, lymphatic invasion, distant metastasis and advanced TNM stage (Table S3). Furthermore, the Kaplan-Meier analysis displayed a relationship between higher MTA2TR mRNA levels and decreased overall survival (OS) (Figure 10A). Moreover, there was a positive correlation between MTA2TR and MTA2 expression (Figure 10B). Coincidently, survival data of TCGA from OncoLnc showed that PC patients with high MTA2 expression had a shorter survival time (Figure 10C). In agreement, the co-staining fluorescence assay further showed an increased MTA2TR accompanied with increased HIF-1α protein expression in PC tissue compared with NP tissue (Figure 10D). TCGA data from ChIPBase revealed HIF-1α expression to be positively correlated with that of MTA2 in PC (Figure 10E). Taken together, these results intensively imply the hypoxia-induced HIF-1α/MTA2TR/MTA2 axis promotes PC development. Thereafter, we depicted a schematic diagram to depict MTA2TR played a vital role in regulation of HIF-1α/MTA2 feedback during hypoxia stress (Figure 10F).

## Discussion

LncRNAs are known to play diverse functions in the initiation and progression of cancers, while the lncRNAs that are specifically related to PC are not fully clarified yet. Here, we revealed MTA2TR is overexpressed in PC tissues, which promotes PC cell proliferation and invasion in vitro and in vivo. In addition, overexpression of MTA2TR is associated with malignant characteristics and short overall survival. It is therefore very important to explore the molecular mechanisms linking MTA2TR with PC tumorigenesis.

Since MTA2TR is adjacent to MTA2, one of the pivotal metastasis-associated protein in numerous cancers, we firstly presumed that whether MTA2 is a downstream target of MTA2TR to exert promoting effects in PC. MTA2 is one of the MTA family members, which form a key part of the nucleosome remodeling and deacetylase (NuRD) complex 28, thus regulating networks of gene expression globally 29, 30. An analysis of MTA2 DNA binding sites found that the actin cytoskeleton, adhere junction, tight junction, and focal adhesion pathways were all enriched and potentially regulated by MTA2 31, 32. Upregulation of MTA2 was revealed in many cancers, such as cervical cancer, hepatocellular carcinoma, pancreatic ductal adenocarcinoma, contributing an increasingly aggressive phenotype 33. In gastric cancer, transcription factor specificity protein 1 (Sp1) regulates the transcriptional level of MTA2 via binding to its promoter 34. One result from Zhao et al. demonstrated that lncRNA-SNHG5 disrupts the progression of gastric cancer progression via constraining MTA2 within the cytosol, which indicating the correlation of MTA2 with lncRNAs 35. Specifically, our research demonstrated MTA2 is regulated by MTA2TR in transcriptional level. On the other hand, the MTA2 overexpression rescued the inhibiting effects of MTA2TR depletion, while MTA2 knockdown successfully reversed the promotion of MTA2TR in PC cells. Moreover, PC samples demonstrated a significant association between MTA2 and MTA2TR. Therefore, MTA2 is thus a key target of MTA2TR in PC. In line with previous reports showed that MTA2 is a biomarker for the poor prognosis of PC 36, we also validate MTA2 is a critical oncogenic driver for growth and metastasis of PC.

We further sought out to figure out the mechanism for the MTA2TR regulating on MTA2 expression. Intersection analysis of TCGA, JASPAR and catRAPID database indicated ATF3 might be an MTA2TR-associated transcription factor, which is required for the regulation of MTA2 expression. ATF3 is one member of the ATF family of basic-region leucine zipper (bZIP) transcription factors. Ectopically expressing ATF3 can mediate the expression of markers of the epithelial-to-mesenchymal transition (EMT) in breast cancer cells 37. In our present study, we identified that ATF3 acts as a transcription factor, which is engaged by MTA2TR to activate MTA2 transcription. Moreover, the gain- and loss-function experiments further verified the tumor promoting effect of ATF3 in PC that is critical for oncogenic function of MTA2TR. Therefore, our data supply new data about how ATF3 functions as oncogene in PC. In contrast, some evidence suggests ATF3 might be an inhibitor in tumorigenesis. Research demonstrated the loss of ATF3 promotes prostate cancer progression of in PTEN knockout mice through activating AKT pathway 38. Similarly, KLF6 induces ATF3 overexpression promotes apoptosis in human prostate cancer cells 39. Moreover, NSAIDs increased ATF3 expression and consequently repressed invasive ability of human colorectal cancer cells 40. In addition, these results suggest the role of ATF3 is heterogeneous in different cancer cell content. Therefore, the identification of ATF3 targets represents an important means of accurately understanding how this protein contributes to cancer.

Hypoxia is a generally universal feature of solid tumors with important implications for tumor treatment and progression 41, 42. A large amount of ongoing research is aimed at exploring how hypoxia-responsive gene networks affect cancer progression 43, 44. There are many studies detecting disrupted lncRNA expression in many cancers under conditions of hypoxia, suggesting that lncRNAs play a role in the hypoxia response. LncRNA-AK123072 was induced under hypoxia and promoted migration and invasion of gastric cancer cells by upregulating EGFR 45. Similarly, our previous study revealed that lncRNA-NUTF2P3-001 is overexpressed during hypoxia and promote PC tumorigenesis via through upregulating K-ras expression. In addition to proteasomal degradation via the VHL ubiquitination complex, recent research has also demonstrated that the stability of HIF-1α is further regulated by acetylation which is mediated by ARD1 46 or Histone deacetylases (HDACs) 47. Previous research has shown that MTA2 deacetylates the estrogen receptor alpha and p53 proteins, thereby suppressing their transcriptional activation 48, 49. Moreover, our previous data indicated MTA2 deacetylates the HIF-1α protein, enhancing its transcriptional activity of HIF-1α 26. As MTA2TR regulates MTA2 expression, it was therefore of interest to assess whether MTA2TR was involved in the stabilization of HIF-1α protein under hypoxic conditions. We found that MTA2TR promoted the association of HIF-1α with MTA2, thereby inducing HIF-1α deacetylation and accumulation of HIF-1α during hypoxia. Moreover, the transcriptional activity of HIF-1α in PC cells under hypoxic conditions was significantly enhanced by MTA2TR. Due to potential HRE in the MTA2TR promoter area, we further investigated the whether MTA2TR is regulated by HIF-1α. Interestingly, our data revealed that hypoxia markably enhanced HIF-1α binding to the MTA2TR promoter, which consequently induced MTA2TR transcription. Together, these results indicate a reciprocal feedback mechanism between MTA2TR and HIF-1α in PC cells under hypoxic conditions. Similarly, work by Yang F.et al has demonstrated that the hypoxia-responsive lincRNA-p21 is required for hypoxia-enhanced glycolysis by enhancing stabilization of HIF-1α 50.

## Conclusions

The present study revealed a reciprocal feedback of HIF-1α and MTA2TR drives hypoxia-induced tumorigenesis of PC, and suggest MTA2TR may be a promising therapeutic target for PC treatment.

## Supplementary Material

Supplementary figures and tables.

## Abbreviations

HIF-1α: Hypoxia-induced factor 1alpha
ceRNA: competitive endogenous RNA
PC: pancreatic cancer
NP: noncancerous peritumoral
lncRNA-MTA2TR: long none coding RNA - MTA2 transcriptional regulator
MTA2: metastasis-associated protein 2
ATF3: activating transcription factor 3
FBS: fetal bovine serum
CHX: Cycloheximide
NCCN: National Comprehensive Cancer Network
RT-PCR: Reverse transcription-PCR
qRT-PCR: quantitative real-time RT-PCR
ChIP: Chromatin immunoprecipitation
RNA-FISH: RNA-Fluorescence in situ hybridization
RIP: RNA immunoprecipitation
HPDE: human pancreatic ductal epithelial cells
WT: wild-type
MUT: mutant binding sites
HREs: hypoxia responsive elements
IHC: Immunohistochemical
LV: lentivirus
NuRD: nucleosome remodeling and deacetylase
Sp1: specificity protein 1
bZIP: basic-region leucine zipper
EMT: epithelial-to-mesenchymal transition
