# Supplementary material for: LncRNA-MTA2TR functions as a promoter in pancreatic cancer via driving deacetylation-dependent accumulation of HIF-1α
Source: Theranostics. 2019 Jul 9;9(18):5298–314. doi: 10.7150/thno.34559 (PMC6691583; doi:10.7150/thno.34559)
Supplement: Supplementary file 1 — Supplementary figures and tables. [file thnov09p5298s1.pdf]

Supplementary Table S1

PC downregulation												
**ProbeName**	**P-value**	**Absolute Fold change([PC] vs [NP])**	**Regulation([PC] vs [NP])**	**type**	**seqname**	**GeneSymbol**	**source**	**RNAlength**	**chrom**	**strand**	**txStart**	**txEnd**
ASHG19A3A037427	0.017751341	2.107672264	down	noncoding	ENST00000414355	CR848007.6	Ensembl	343	chr9	-	44021071	44021414
ASHG19A3A032810	0.002665768	2.835939981	down	noncoding	ENST00000434161	CTD-2021A8.3	Ensembl	423	chr7	-	51456093	51457401
ASHG19A3A044751	0.020793253	2.324668015	down	noncoding	ENST00000449749	AC068580.7	Ensembl	338	chr11	-	1798159	1799618
ASHG19A3A043972	0.04265114	2.049745083	down	noncoding	ENST00000435086	RP11-448K10.1	Ensembl	1884	chr10	+	59715054	59716938
ASHG19A3A029793	0.021125308	2.111288162	down	noncoding	ENST00000501646	AL662799.2	Ensembl	1912	chr6	-	33395516	33421465
ASHG19A3A035090	0.00300107	3.241126883	down	noncoding	ENST00000445459	AC018643.4	Ensembl	744	chr7	+	131945619	131956028
ASHG19A3A046696	0.007190716	2.200407156	down	noncoding	ENST00000429268	AP001271.4	Ensembl	356	chr11	+	70492269	70493667
ASHG19A3A018758	0.020736914	2.367789453	down	noncoding	ENST00000406413	AP000567.27	Ensembl	156	chr21	-	31898863	31899019
ASHG19A3A032454	0.010936227	2.24831667	down	noncoding	ENST00000342275	RP11-740N7.1	Ensembl	897	chr7	-	6874471	6920187
ASHG19A3A032544	0.020695405	2.008721563	down	noncoding	ENST00000421730	AC005682.8	Ensembl	675	chr7	-	22928989	22980809

PC upregulation												
ASHG19A3A026824	0.029096242	2.34710283	up	noncoding	ENST00000510053	CTD-2516K3.3	Ensembl	2025	chr5	-	56613815	56615847
ASHG19A3A051446	0.001376558	2.439985797	up	noncoding	ENST00000254302	UBE2L7P	Ensembl	465	chr14	+	55695934	55696399
ASHG19A3A015537	0.006877329	2.84426749	up	noncoding	ENST00000395092	GAPDHP25	Ensembl	1002	chr2	+	38512551	38513558
ASHG19A3A053659	0.010127734	2.968233319	up	noncoding	ENST00000379752	RP11-110J1.2	Ensembl	1532	chr1	-	157029022	157030554
ASHG19A3A044034	0.013576093	2.389886328	up	noncoding	ENST00000415623	RP11-9E13.4	Ensembl	438	chr10	+	70184316	70184754
ASHG19A3A034357	0.008079381	2.006102471	up	noncoding	ENST00000434745	RP11-613E4.2	Ensembl	2818	chr7	+	55804471	55807289
ASHG19A3A044659	0.001200056	2.076656357	up	noncoding	ENST00000417351	CTAGE7	Ensembl	2823	chr10	+	131904272	131907095
ASHG19A3A030715	0.000940944	2.221480535	up	noncoding	ENST00000405959	RP1-101K10.4	Ensembl	362	chr6	-	153280411	153280773
ASHG19A3A038809	0.029258824	2.05208857	up	noncoding	ENST00000451550	ATP5J2P3	Ensembl	258	chr9	+	79655331	79655589
MTA2TR	0.036418382	2.1795511	up	misc_RNA				158	chr11	+	62321164	62321291

Supplementary Table S2

Coding Potential Calculator

HOME RUN CPC DOCUMENTS CONTACT

quick links

- Run CPC
- Get Results
- Quick Guide
- Download
- Documents

This is the **Table View** of CPC results. Your task ID is **B0214B10-5733-11E7-9E0D-B99392AAE7A3** . You may use the ID to retrieve your results later in our view retrieve page . You can also view **Raw Data** of the CPC results.

ID	C/NC	CODING POTENTIAL SCORE	EVIDENCE	UTR-DB HITs	RNA-DB HITs
AF083120.1	noncoding (weak)	-0.442783	detail	search	search

view filter

show coding status: all ; score range: : ; sort by: SCORE refresh

Home · Run CPC · Documents · Contact

Copyright © 2006 - 2015 Center for Bioinformatics, Peking University. All rights reserved.

CPAT
Coding Potential Assessment Tool

Calculator User Guide Feedback Source Code

Result for species name : hg19 with job ID :1483842968							
Data ID	Sequence Name	RNA Size	ORF Size	Ficket Score	Hexamer Score	Coding Probability	Coding Label
0	AF083120.1	158	60	0.7756	-0.192355461921	0.0018496507672512	no

This job has been stored with the job ID
Download Table in tab delimetered file (.txt)

For suggestions, comments or queries about this website,
please leave your feedback through Feedback.
Copyright © 2012. All rights reserved.

BCM Baylor College of Medicine

MAYO CLINIC

Supplementary Table S3: Correlation between overexpressed lncRNA-MTA2TR and clinical characteristics of patient with pancreatic cancer

Characteristics	Number of cases	MTA2TR expression		P value
		High	low	
Total cases	40			
Gender				
Male	22	10	12	0.3239
Female	18	11	7	
Age				
<60	20	13	7	0.7440
≥60	20	12	8	
Tumor size(cm)				
<2	18	13	5	0.011*
≥2	22	7	15	
Histological Grade				
High/Moderate	21	14	7	0.0267*
Low	19	6	13	
Lymphatic invasion				
Positive	25	17	8	0.0329*
Negative	15	5	10	
Vascular invasion				
Positive	24	13	11	0.5186
Negative	16	7	9	
Distant Metastasis				
Positive	21	16	5	0.0120*
Negative	19	7	12	

Note: Overexpression of lncRNA-MTA2TR was signifcantly associated with large tumor size (P = 0.011), poor tumor differentiation (P = 0.0222), lymphatic invasion (P = 0.0329), distant metastasis (P = 0.0120) and TNM stage (P = 0.0267), but not with patients' age (P = 0.744), gender (P = 0.3239) and vascular infltration P = 0.5186). The p-value represents the comparison between groups (*p < 0.05, **p < 0.01).

Supplementary Table S4. The sequence of PCR primers.

Primer	Sequence
MTA2TR	Forward: 5’-CACTTCCACGCTCCTTACCA-3’
	Reverse: 5’-GAGCGAGGCATCTAAAGGCT-3’
MTA2	Forward: 5’-GGCGCAGGGACATTTCTAGT-3’
	Reverse: 5’-AGAAGTGTCTTCTGCACGGG-3’
preMTA2	Forward: 5’-CAAGCGTCAGAAACTAAACC-3’
	Reverse: 5’-GAAAGAGCAAGTGGGAAAC-3’
ATF3	Forward: 5’-CTAAGCAGTCGTGGTATGG-3’
	Reverse: 5’-TGGAGTTGAGGCAAAGAT-3’
GAPDH	Forward: 5’-TGAACGGGAAGCTCACTGG-3’
	Reverse: 5’-TCCACCACCCTGTTGCTGTA-3’
β-actin	Forward: 5’-CATGTACGTTGCTATCCAGGC-3’
	Reverse: 5’-CTCCTTAATGTCACGCACGAT-3’
MTA2 CHIP Primer	Forward: 5’-GGTCACTCCAGCTTCATCACT-3’<br>Reverse: 5’-ACAGCTTCCACGAGTTCCTC-3’
HIF-1α	Forward: 5′-CAAGATCTCGGCGAAGCAA-3′
	Reverse: 5′-GGTGAGCCTCATAACAGAAGCTTT-3′
MTA2TR CHIP	Forward: 5’-TCCACCACCATTCAGAGG-3’
Primer1	Reverse: 5’-GAGCCCAAGGTTCATTACAGACGCG-3’
Primer2	Forward: 5’-CCAGAAGGCAAATGAAGT-3’
	Reverse: 5’-GATGGTGCTAAGAAGGGTT-3’
Primer3	Forward: 5’-TTACAGTTCCCTAAGTCCAGTT-3’
	Reverse: 5’-TCAGATGTAGCGATCCAAGT-3’
MTA2TR FISH (Northern blot)	Forward:5’-TAATACGACTCACTATAGGGGGTAGGGTTGCCAAGGT-3’<br>Reverse:5’-CAGGGCTGGCTCCACTTCCACGCTC-3’
MTA2TR RNA Pull down	Forward:5’-TAATACGACTCACTATAGGGCGGGGGGGAGGAGCCAG CCCTGGCCCCAGGGCTGGCTCCACTT-3’
	Reverse:5’-GGTAGGGTTGCCAAGGTGGACACAGCATACCAA-3’
VEGFa	Forward:5’-CATCTTCAAGCCATCCTGTGTG-3’
	Reverse:5’- CCGCATAATCTGCATGGTGAT-3’
VEGFb	Forward:5’-GAGATGTCCCTGGAAGAACACA-3’
	Reverse:5’-GAGTGGGATGGGTGATGTCAG-3’
LOX	Forward:5’-CGGCGGAGGAAAACTGTCT-3’
	Reverse:5’-TCGGCTGGGTAAGAAATCTGA-3’
LOXL2	Forward:5’-GGGTGGAGGTGTACTATGATGG-3’
	Reverse:5’-CTTGCCGTAGGAGGAGCTG-3’
PLOD2	Forward:5’-CATGGACACAGGATAATGGCTG-3’
	Reverse:5’-AGGGGTTGGTTGCTCAATAAAAA-3’
TUBA1A	Forward:5’-TCGATATTGAGCGTCCAACCT-3’
	Reverse:5’-CAAAGGCACGTTTGGCATACA-3’

Supplementary Table S5. The sequence of siRNA.

siRNA	Sequence
NC-siRNA	Sense: 5′-UUCUCCGAACGUGUCACGUTT-3′
(siNC)	Antisense: 5′-ACGUGACACGUUCGGAGAATT-3′
CATX-4-homo-37	Sense: 5′-UCCACUUCCACGCUCCUUATT-3′
(siMTA2TR#1)	Antisense: 5′-UAAGGAGCGUGGAAGUGGATT-3′
CATX-4-homo-86	Sense: 5′-GGAAGCCUUUAGAUGCCUCTT-3′
(siMTA2TR#2)	Antisense: 5′- GAGGCAUCUAAAGGCUUCCTT-3′
MTA2-homo-814	Sense: 5′-GCUGAUCAGGGCGAGAUUATT-3′
(siMTA2#1)	Antisense: 5′-UAAUCUCGCCCUGAUCAGCTT-3′
MTA2-homo-2364	Sense: 5′-GCACCAAUGAGCCUAUUGUTT-3′
(siMTA2#2)	Antisense: 5′- ACAAUAGGCUCAUUGGUGCTT-3′
MTA2-homo-1400	Sense: 5′-CCACCUACACUAAGCCAAATT-3′
(siMTA2#3)	Antisense: 5′-UUUGGCUUAGUGUAGGUGGTT-3′
ATF3-homo-444	Sense: 5′-GGUUUGCCAUCCAGAACAATT-3′
(siATF3#1)	Antisense: 5′-UUGUUCUGGAUGGCAAACCTT-3′
ATF3-homo-605	Sense: 5′-GCUGCAAAGUGCCGAAACATT-3′
(siATF3#2)	Antisense: 5′-UGUUUCGGCACUUUGCAGCTT-3′
ATF3-homo-811	Sense: 5′-CCUCUUUAUCCAACAGAUATT-3′
(siATF3#3)	Antisense: 5′-UAUCUGUUGGAUAAAGAGGTT-3′
HIF1α-homo-964	Sense: 5′-GCUGAUUUGUGAACCCAUUTT-3′
(siHIF1α#1)	Antisense: 5′-AAUGGGUUCACAAAUCAGCTT-3′
HIF1α-homo-1116	Sense: 5′-GCCGCUCAAUUUAUGAAUATT-3′
(siHIF1α#2)	Antisense: 5′-UAUUCAUAAAUUGAGCGGCTT-3′
HIF1α-homo-1556	Sense: 5′-GCCUCUUUGACAAACUUAATT-3′
(siHIF1α#3)	Antisense: 5′-UUAAGUUUGUCAAAGAGGCTT-3′

LV-siMTA2TR#1, LV-siMTA2TR#2

Carrier Name: GV248

Component sequence: hU6-MCS-Ubiquitin-EGFP-IRES-puromycin

Control number: CON077

Control insert sequence: TTCTCCGAACGTGTCACGT

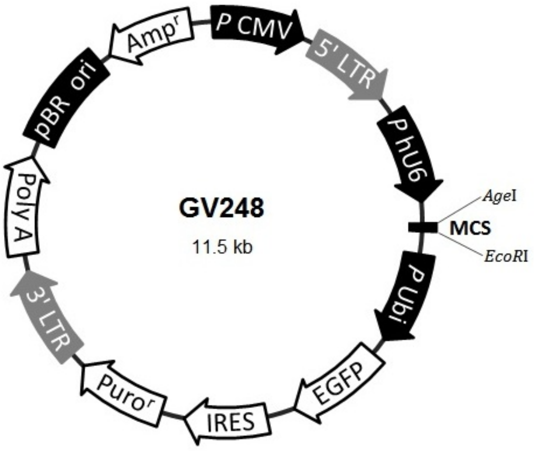

MTA2TR-OE
Carrier Name: GV144
Component sequence: CMV-EGFP-MCS-SV40-Neomycin
Cloning site: XhoI / BamHI
Target sequence: CTTGACGGGGGGGAGGAGCCAGCCCTGGCCCCAGGGCTG GCTCCACTTCCACGCTCCTTACCAAGCTACCCTTACCAAGCTACGGGTGCA GGAAGCCTTTAGATGCCTCGCTCTCCCTGCCCCTCCACACTTGGTATGCTGT GTCCACCTTGGCAACCCTACC
Control Number: CON107

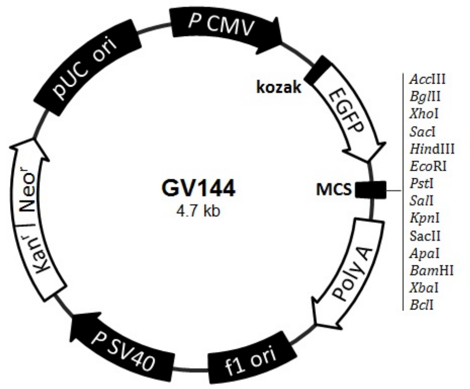

Supplementary Figure Legends

Figure S1. MTA2TR facilitates the in vivo invasion and proliferation of SW1990 cells. (A-F) SW1990 cells transfected using lentiviruses encoding siMTA2TR#1 (LV-siMTA2TR#1), siMTA2TR#2 (LV-siMTA2TR#2) or siNC (LV-siNC) were transplanted subcutaneously into nude mice. **(A)** In Vivo Fluorescence Imaging demonstrated the subcutaneous tumor. **(B)** The weight of subcutaneous tumor was measured after 4 weeks when mice were sacrificed. **(C)** The volume of subcutaneous tumor was measured every 4 days. **(D)** MTA2TR expression was compared in the subcutaneous tumor form LV-siMTA2TR#1, LV-siMTA2TR#2 or LV-siNC group. **(E)** MTA2 mRNA expression was detected in the subcutaneous tumor form LV-siMTA2TR#1, LV-siMTA2TR#2 or LV-siNC group. **(F)** The MTA2 protein expression was demonstrated by IHC in the subcutaneous tumor form LV-siMTA2TR#1, LV-siMTA2TR#2 or LV-siNC group. **(G-K)** SW1990 cells transfected with LV-siMTA2TR#1, LV-siMTA2TR#2 or LV-siNC were transplanted via tail vein injection to observe tumor metastasis. **(G)** In Vivo Fluorescence Imaging indicated the metastasis of SW1990 cells in LV-siMTA2TR#1, LV-siMTA2TR#2 or LV-siNC group when mice were sacrificed after 4 weeks. Arrows indicate the invasion nodules. Scale bars, 1 cm. **(H)** Liver metastasis was measured with the indicated SW1990 cells. N=5 mice in each group. **(I)** The number of visible liver metastases per 5 sections in each nude mouse. **(J, K)** H&E images of liver and lung tissue isolated from LV-siMTA2TR#1, LV-siMTA2TR#2 or LV-siNC group. Arrows indicate the invasion nodules. Scale bars, 100 μm.

Figure S2. MTA2TR promotes proliferation and invasion of PC cells. (A) Stable overexpression of MTA2TR was detected in BxPC-3/SW1990 cells using qRT-PCR after transfection of control Vector (Vector) or MTA2TR overexpression vector (MTA2TR-OE). **(B, C)** The effect of MTA2TR overexpression on BxPC-3/SW1990 cells proliferations was measured by MTT and colony formation assay respectively. **(D, E)** The effect of MTA2TR overexpression on invasion and migration was assessed by transwell and wound healing assays in BxPC-3/SW1990 cells respectively.

Figure S3. MTA2TR predominantly functions through regulating MTA2. (A-C)

BxPC-3/SW1990 cells were transfected with control, siMTA2TR#1, siMTA2TR#2, or/and MTA2-OE. **(A)** The mRNA and protein levels of MTA2 were detected by qRT-PCR and western blot in transfected BxPC-3/SW1990 cells. **(B)** Cell proliferations were measured by MTT assay in transfected BxPC-3/SW1990 cells. **(C)** Assessment of invasion was measured by transwell assay in transfected BxPC-3/SW1990 cells. The right histogram represents relative cell number while the representative images are shown on the left panel. Average counts from five random microscopic fields.

Figure S4. MTA2TR predominantly functions by regulating MTA2. (A-C) BxPC-3/SW1990 cells were transfected with control, MTA2TR-OE, siMTA2#1, or/and siMTA2#2. **(A)** The mRNA and protein levels of MTA2 were detected by qRT-PCR and western blot in transfected BxPC-3/SW1990 cells. **(B)** Cell proliferations were measured by MTT assay in transfected BxPC-3/SW1990 cells. **(C)** Assessment of invasion was measured by transwell assay in transfected BxPC-3/SW1990 cells. The right histogram represents relative cell number while the representative images are shown on the left panel. Average counts from five random microscopic fields.

Figure S5. MTA2TR activates transcription of MTA2 by recruiting ATF3 to the MTA2 promoter. (A) The efficiency of ATF3 knockdown on BxPC-3/SW1990 cells was evaluated by qRT-PCR and western blot. **(B)** The effect of ATF3 overexpression on ATF3 expression of BxPC-3/ SW1990 cells was evaluated by qRT-PCR and western blot. **(C, D)** BxPC-3 cells were transfected with control, siMTA2TR#1 or and ATF3-OE. **(C)** The binding between ATF3 and MTA2 promoter in the transfected BxPC-3 cells was evaluated by ChIP assay. **(D)** The transfected BxPC-3 cells were co-transfected with vector containing WT or MUT of MTA2 promoter as reporter cells. The promoter activity of these reporter cells was analyzed by luciferase reporter assay. **(E)** BxPC-3/SW1990 cells were transfected with control, siMTA2TR#1, siMTA2TR#2 or and ATF3-OE. The mRNA and protein level of MTA2 was detected in transfected BxPC-3/SW1990 cells.

Figure S6. MTA2TR predominantly functions through its association with MTA2 by recruiting ATF3. (A, B) BxPC-3/SW1990 cells were transfected with control, siMTA2TR#1, siMTA2TR#2 or and ATF3-OE. **(A)** Cell proliferations were measured

by MTT assay. **(B)** Assessment of invasion was measured by transwell assay. The right histogram represents relative cell number while the representative images are shown on the left panel. Average counts from five random microscopic fields.

Figure S7. MTA2TR predominantly functions through regulating MTA2 by recruiting ATF3. (A, B) BxPC-3/SW1990 cells were transfected with control, MTA2TR-OE, siATF3#1 or and siATF3#2. **(A)** Cell proliferations were measured by MTT assay. **(B)** Assessment of invasion was measured by transwell assay. The right histogram represents relative cell number while the representative images are shown on the left panel. Average counts from five random microscopic fields.

Figure S8. Hypoxia-Induced MTA2TR increased the recruitment of ATF3 on the MTA2 promoter. (A) The binding between MTA2TR and ATF3 was verified by RIP assay with anti-ATF3 antibody in BxPC-3/SW1990 cells under normal and hypoxia conditions, and the co-precipitated transcript were determined using qRT-PCR. **(B)** Combined immunofluorescence of ATF3 protein (green) and RNA-FISH analysis of MTA2TR (red) in BxPC-3 cells under normoxia and hypoxia environments. Scale bars, 20 μm. **(C)** The effect of hypoxia on binding of ATF3 on MTA2 promoter was inspected by ChIP assay in BxPC-3/SW1990 cells.

Figure S9. Hypoxia-Induced MTA2TR stabilizes HIF-1α by promoting deacetylation of HIF-1α. (A) The mRNA and protein level of HIF-1α was detected in MTA2TR-overexpression BxPC-3/SW1990 cells by qRT-PCR and western blot. **(B)** The effect of MTA2TR overexpression on stabilization of HIF-1α was measured in BxPC-3/SW1990 cells treated with cycloheximide under hypoxia for the indicated periods of time. **(C)** The expression of HIF-1α in the MTA2TR overexpression BxPC-3/cells was detected under hypoxia with or without MG132 treatment. **(D, E)** The acetylation of HIF-1α and the binding between MTA2 and HIF-1α in the MTA2TR-overexpression BxPC-3/SW1990 cells were analyzed in the cell lysates under hypoxia, which were immunoprecipitated with anti-HIF-1α or anti-MTA2 antibody. **(F)** The acetylation of HIF-1α and the binding between MTA2 and HIF-1α in the BxPC-3/SW1990 cells transfected with MTA2TR-OE or/and siMTA2 under hypoxia were analyzed by immunoprecipitation assay with anti-HIF-1α or anti-MTA2 antibody. **(G)**

The mRNA level of VEGFa, VEGFb, LOX, LOXL2, PLOD2 and TUBA1 was detected in MTA2TR-overexpression BxPC-3/SW1990 cells under hypoxia by qRT-PCR.

Figure S10. Hypoxia-Induced MTA2TR promote proliferation and invasion of BxPC-3/SW1990 cells. (A, B) BxPC-3/SW1990 cells were transfected with siNC, siMTA2TR#1 or siMTA2TR#2 under normal or hypoxia condition. **(A)** Cell proliferations were measured by MTT assay. **(B)** Representative images (upper) and number (below) of invasive cells. Average counts from five random microscopic fields.

Figure S1

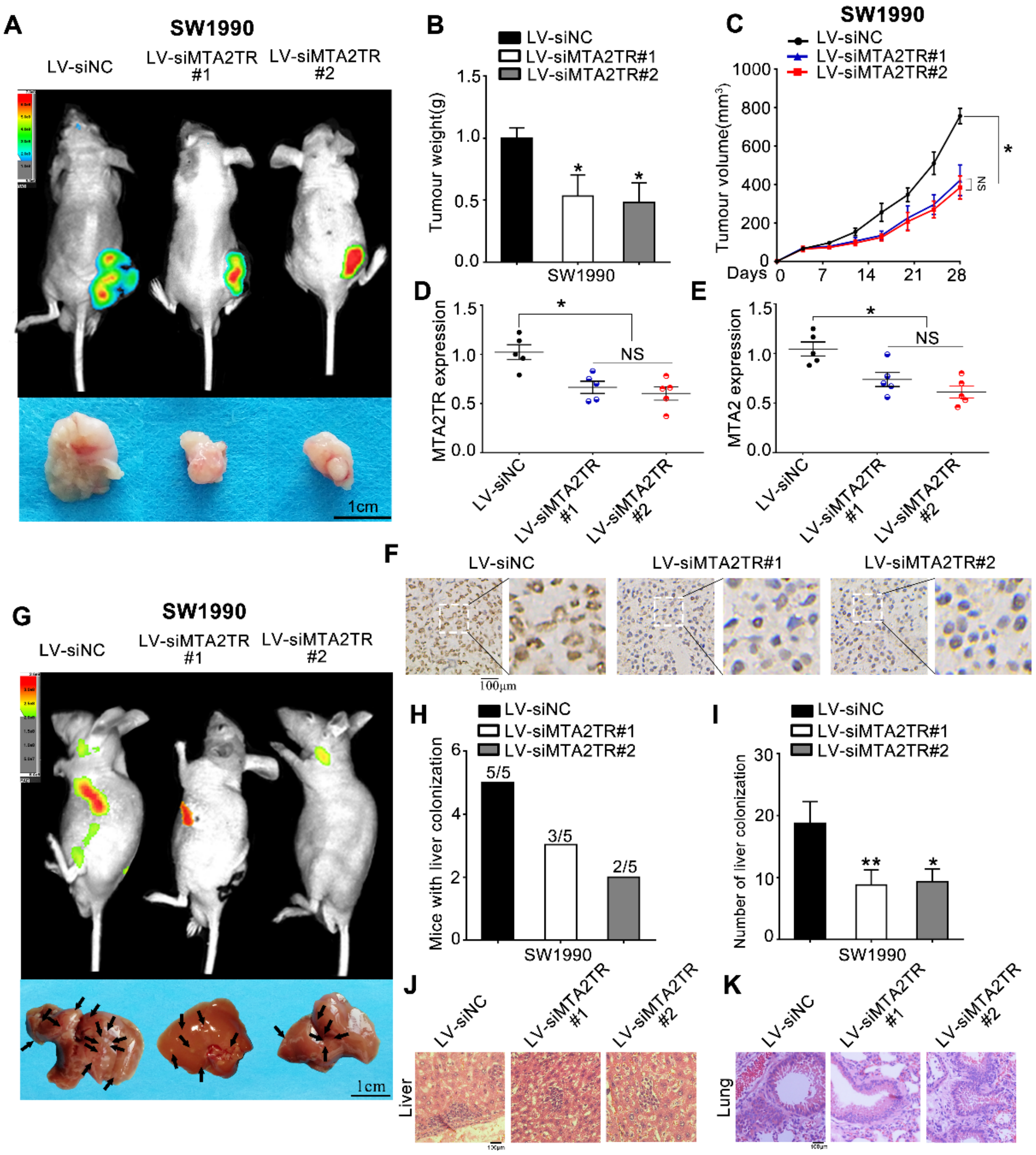
A
SW1990
LV-siNC
LV-siMTA2TR #1
LV-siMTA2TR #2
1cm
B
LV-siNC
LV-siMTA2TR#1
LV-siMTA2TR#2
Tumour weight(g)
SW1990
C
SW1990
LV-siNC
LV-siMTA2TR#1
LV-siMTA2TR#2
Tumour volume(mm3)
Days
NS
D
MTA2TR expression
NS
E
MTA2 expression
NS
F
LV-siNC
LV-siMTA2TR#1
LV-siMTA2TR#2
100μm
G
SW1990
LV-siNC
LV-siMTA2TR #1
LV-siMTA2TR #2
1cm
H
LV-siNC
LV-siMTA2TR#1
LV-siMTA2TR#2
Mice with liver colonization
5/5
3/5
2/5
SW1990
I
LV-siNC
LV-siMTA2TR#1
LV-siMTA2TR#2
Number of liver colonization
SW1990
J
Liver
K
Lung

Figure S2

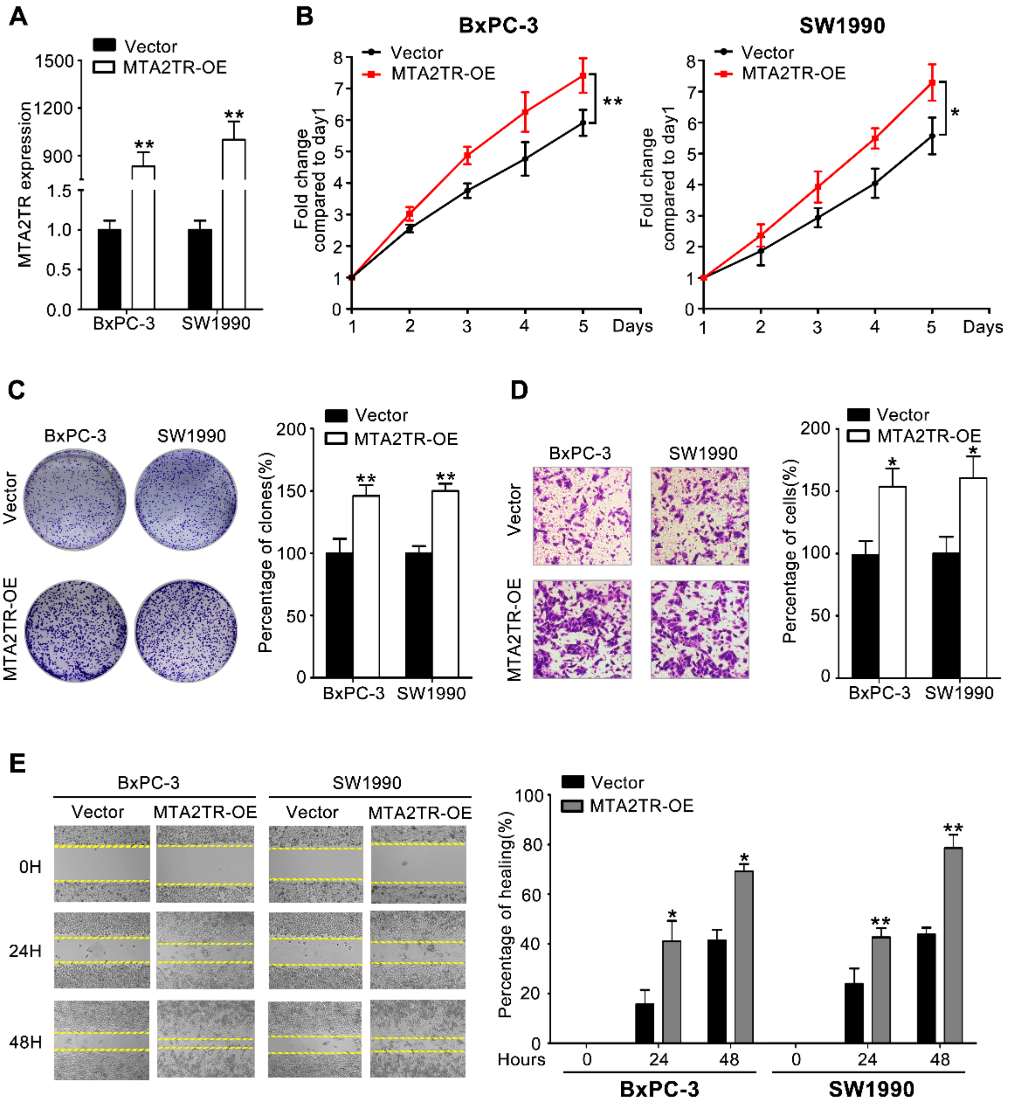
A
Vector
MTA2TR-OE
MTA2TR expression
BxPC-3
SW1990
B
BxPC-3
SW1990
Fold change compared to day1
Days
C
Vector
MTA2TR-OE
Percentage of clones(%)
D
Percentage of cells(%)
E
0H
24H
48H
Percentage of healing(%)
Hours

Figure S3

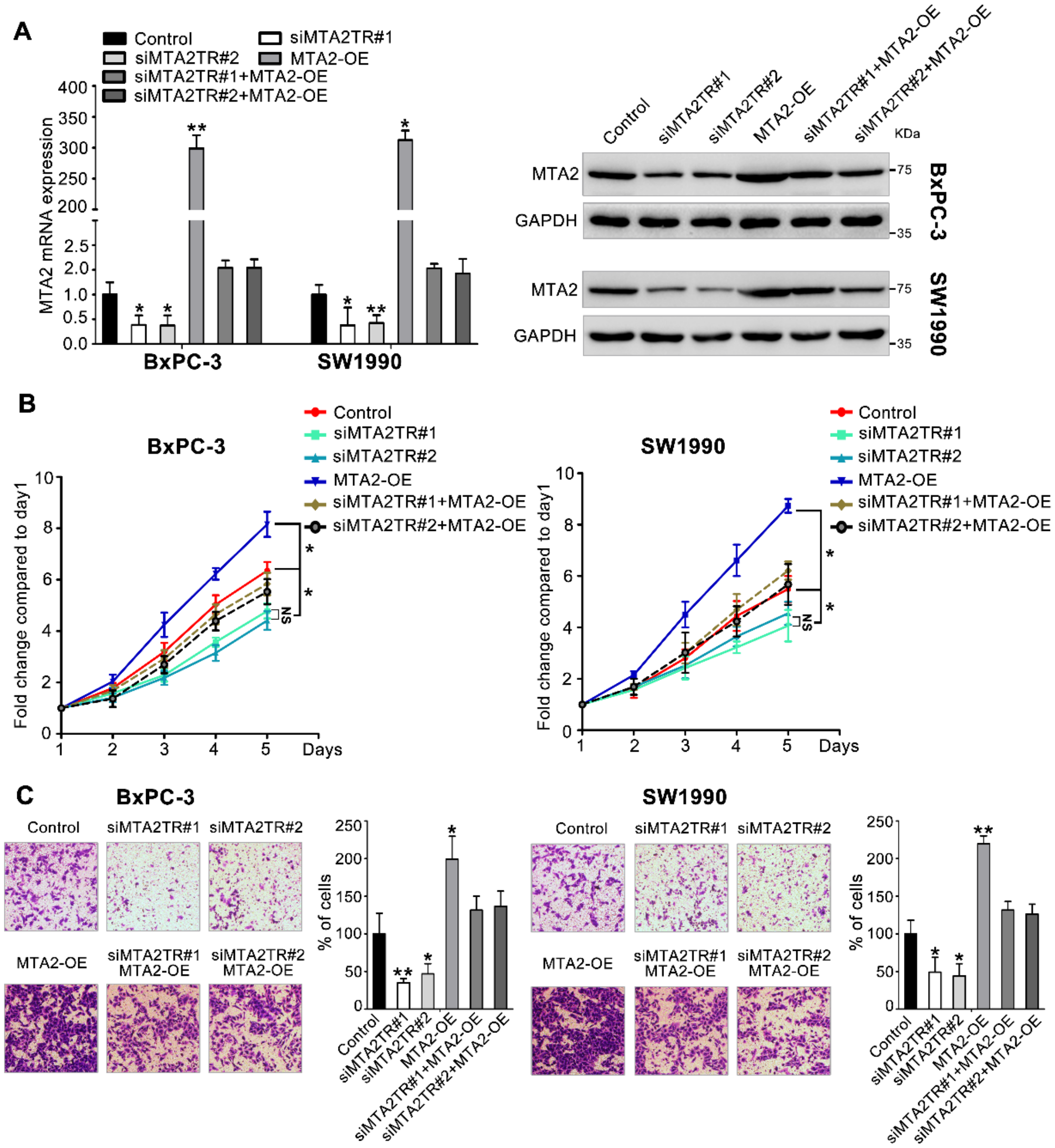

A
Control
siMTA2TR#1
siMTA2TR#2
MTA2-OE
siMTA2TR#1+MTA2-OE
siMTA2TR#2+MTA2-OE
MTA2 mRNA expression
BxPC-3
SW1990
MTA2
GAPDH
KDa
75
35
B
Fold change compared to day1
Days
NS
C
% of cells

Figure S4

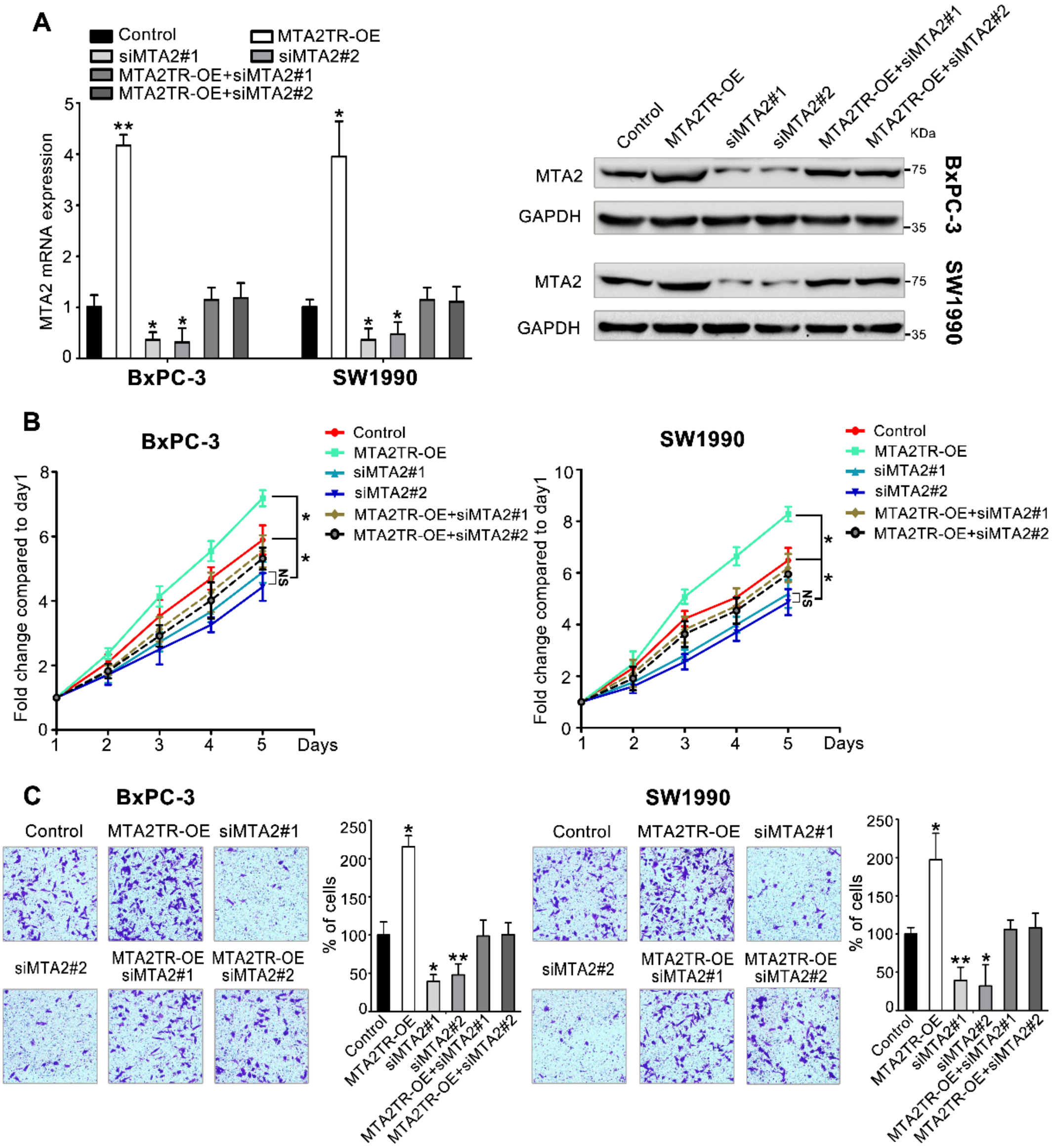
A
Control
MTA2TR-OE
siMTA2#1
siMTA2#2
MTA2TR-OE+siMTA2#1
MTA2TR-OE+siMTA2#2
MTA2 mRNA expression
BxPC-3
SW1990
MTA2
GAPDH
KDa
B
BxPC-3
SW1990
Fold change compared to day1
Days
NS
C
% of cells
MTA2TR-OE siMTA2#1
MTA2TR-OE siMTA2#2

Figure S5

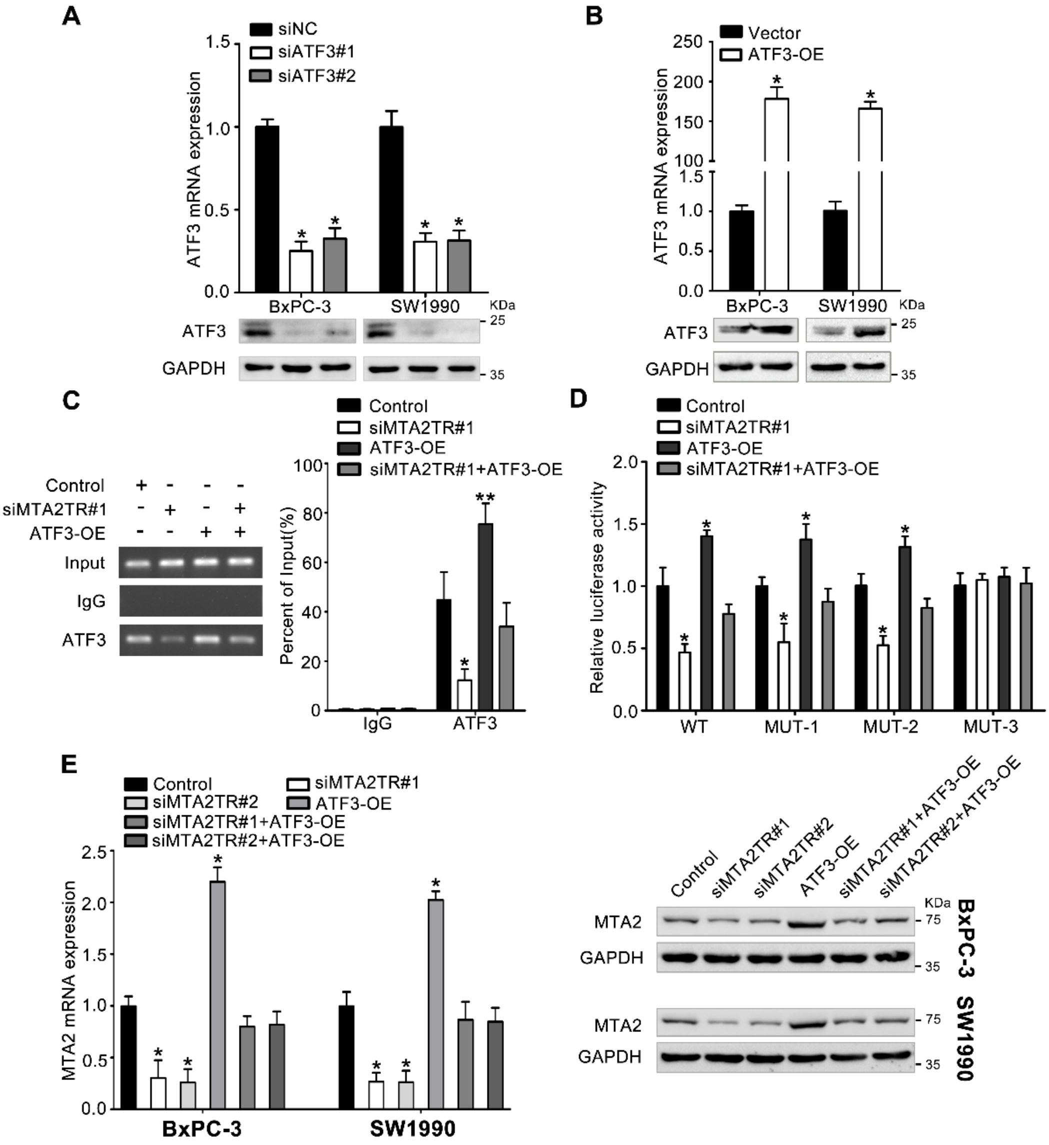
A
siNC
siATF3#1
siATF3#2
ATF3 mRNA expression
BxPC-3
SW1990
ATF3
GAPDH
B
Vector
ATF3-OE
ATF3 mRNA expression
C
Control
siMTA2TR#1
ATF3-OE
siMTA2TR#1+ATF3-OE
Input
IgG
ATF3
Percent of Input(%)
D
Relative luciferase activity
WT
MUT-1
MUT-2
MUT-3
E
siMTA2TR#2
siMTA2TR#2+ATF3-OE
MTA2 mRNA expression
MTA2
KDa

Figure S6

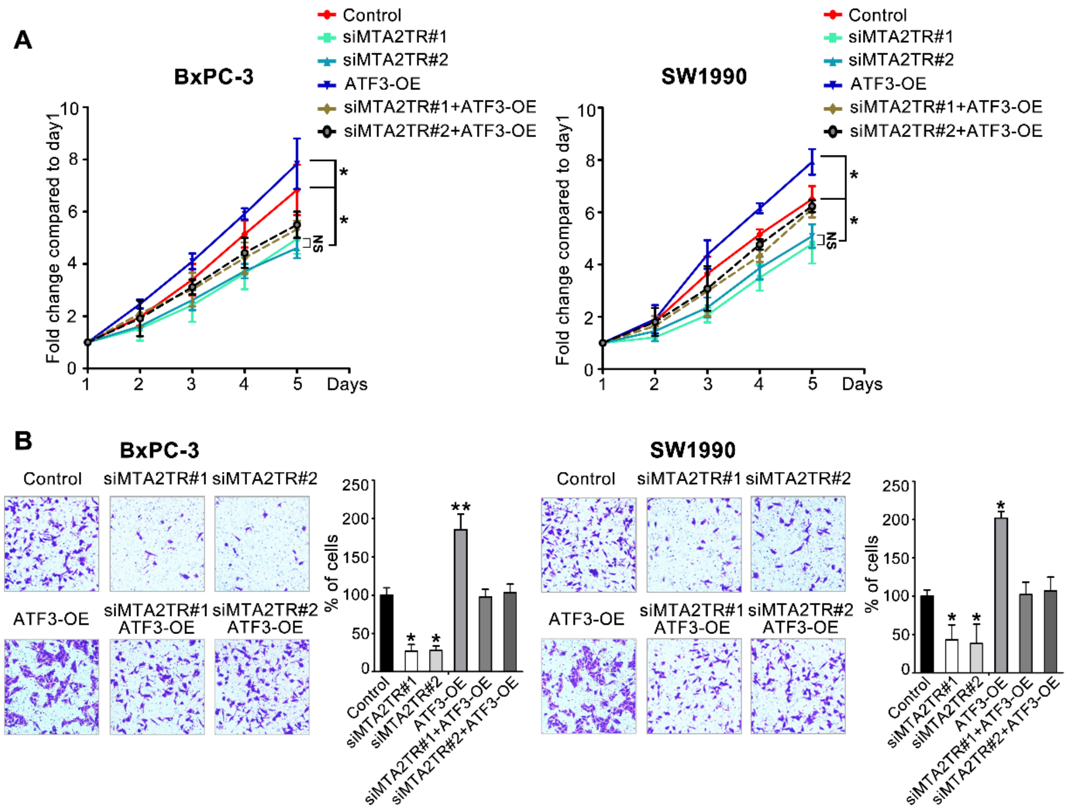

A
BxPC-3
SW1990
Control
siMTA2TR#1
siMTA2TR#2
ATF3-OE
siMTA2TR#1+ATF3-OE
siMTA2TR#2+ATF3-OE
Fold change compared to day1
Days
ns
B
Control
siMTA2TR#1
siMTA2TR#2
ATF3-OE
siMTA2TR#1 ATF3-OE
siMTA2TR#2 ATF3-OE
% of cells

Figure S7

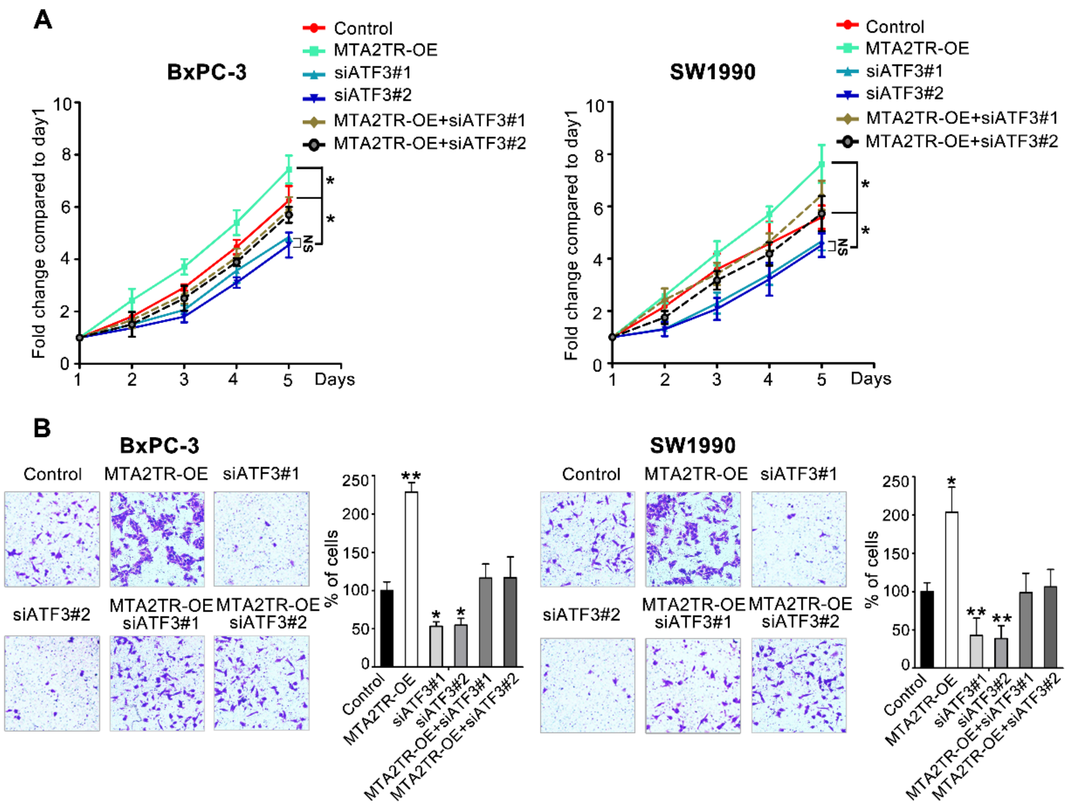
A
BxPC-3
SW1990
Control
MTA2TR-OE
siATF3#1
siATF3#2
MTA2TR-OE+siATF3#1
MTA2TR-OE+siATF3#2
Fold change compared to day1
Days
NS
B
BxPC-3
SW1990
Control
MTA2TR-OE
siATF3#1
siATF3#2
MTA2TR-OE siATF3#1
MTA2TR-OE siATF3#2
% of cells

Figure S8

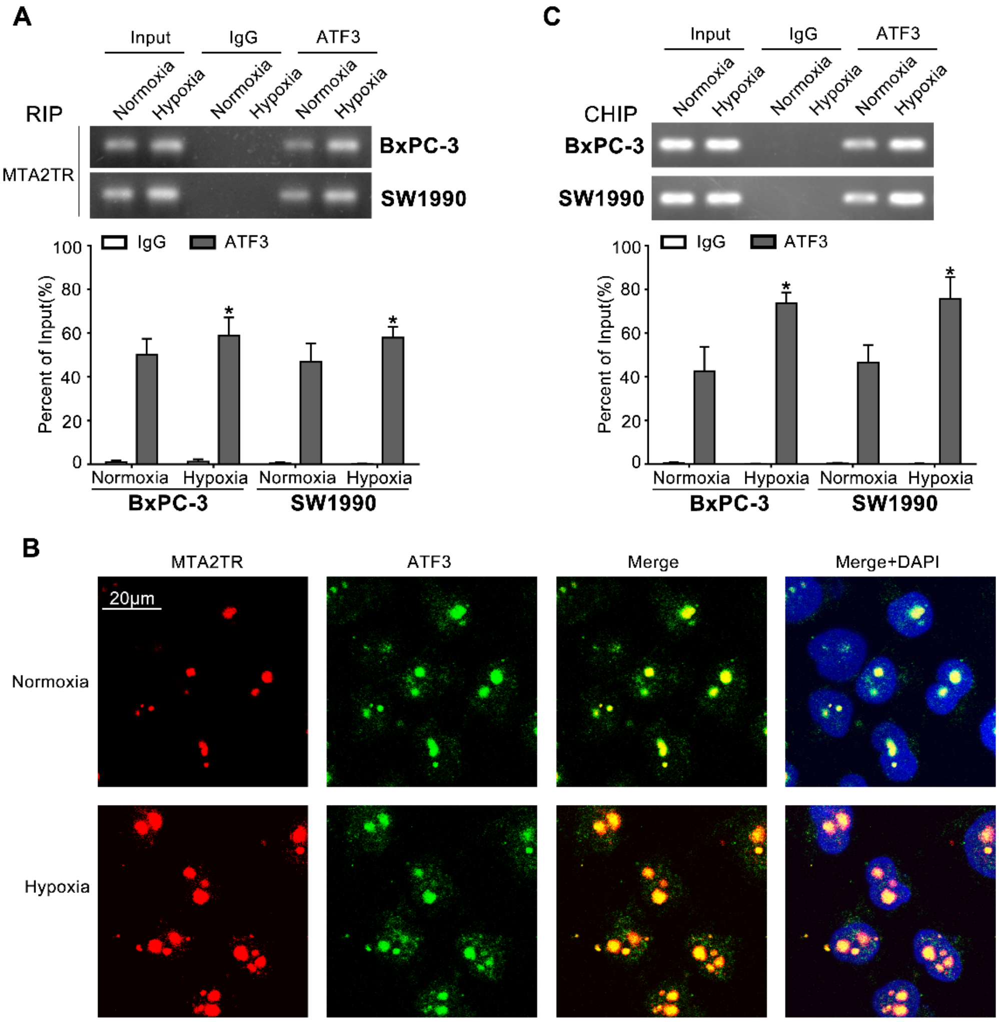

A
Input
IgG
ATF3
Normoxia
Hypoxia
Normoxia
Hypoxia
Normoxia
Hypoxia
RIP
MTA2TR
BxPC-3
SW1990
IgG
ATF3
Percent of Input(%)
100
80
60
40
20
0
Normoxia
Hypoxia
Normoxia
Hypoxia
BxPC-3
SW1990
C
Input
IgG
ATF3
Normoxia
Hypoxia
Normoxia
Hypoxia
Normoxia
Hypoxia
CHIP
BxPC-3
SW1990
IgG
ATF3
Percent of Input(%)
100
80
60
40
20
0
Normoxia
Hypoxia
Normoxia
Hypoxia
BxPC-3
SW1990
B
MTA2TR
ATF3
Merge
Merge+DAPI
20μm
Normoxia
Hypoxia

Figure S9

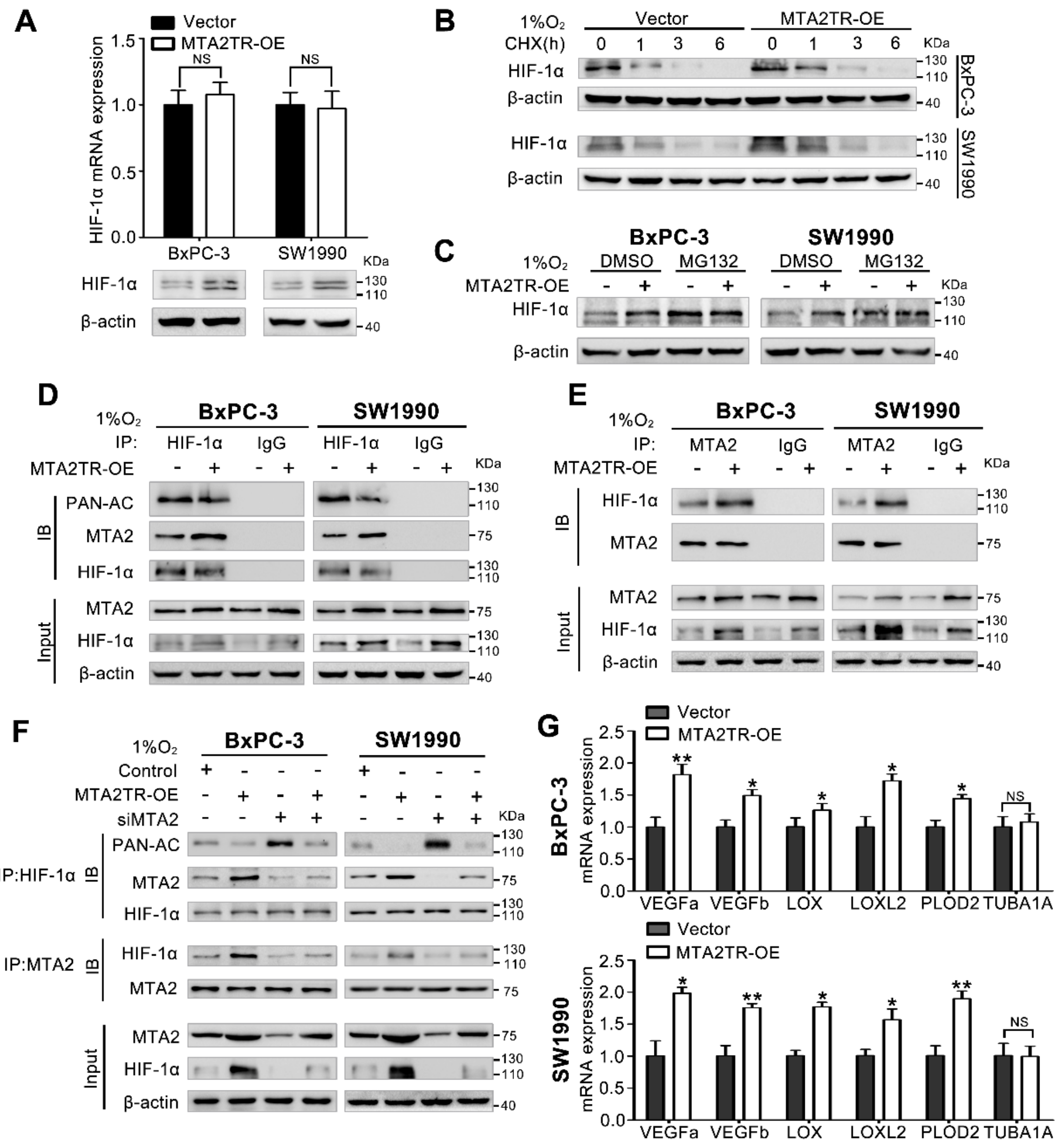

A
Vector
MTA2TR-OE
HIF-1α mRNA expression
NS
BxPC-3
SW1990
HIF-1α
β-actin
KDa
B
1%O2
Vector
MTA2TR-OE
CHX(h)
0 1 3 6 0 1 3 6
HIF-1α
β-actin
BxPC-3
SW1990
C
BxPC-3
SW1990
DMSO
MG132
MTA2TR-OE
D
IP:
HIF-1α
IgG
PAN-AC
MTA2
IB
Input
E
MTA2
F
Control
siMTA2
IP:HIF-1α
IP:MTA2
G
VEGFa VEGFb LOX LOXL2 PLOD2 TUBA1A
mRNA expression

Figure S10

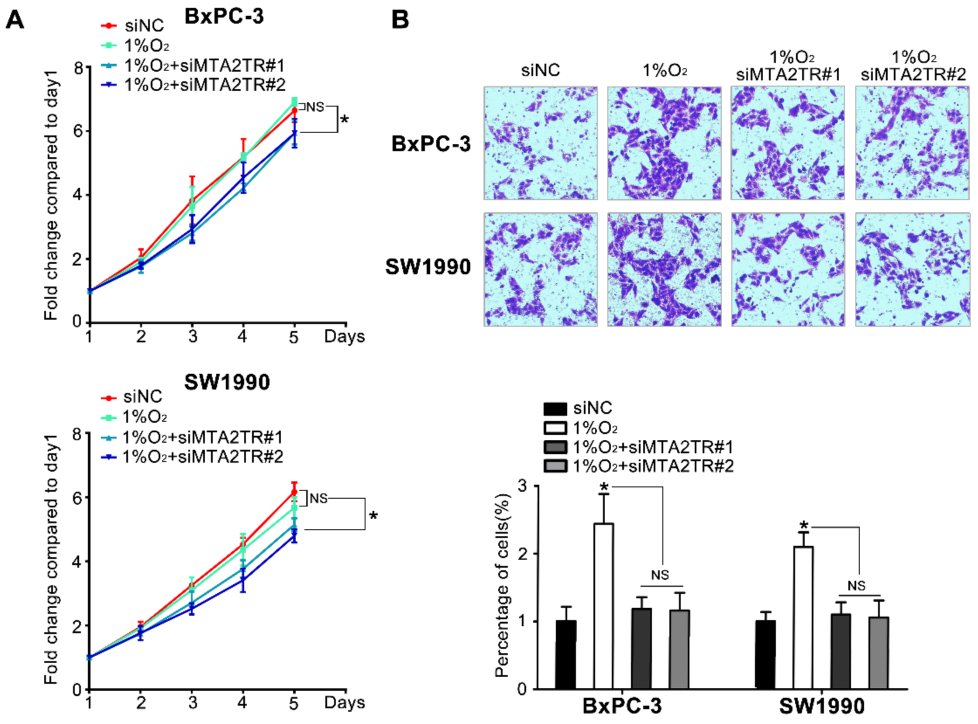
A
BxPC-3
siNC
1%O2
1%O2+siMTA2TR#1
1%O2+siMTA2TR#2
Fold change compared to day1
NS
*
Days
SW1990
B
siNC
1%O2
1%O2 siMTA2TR#1
1%O2 siMTA2TR#2
BxPC-3
SW1990
Percentage of cells(%)
NS

Supplementary Methods

Cell proliferation assay

Cells were plated in 96-well plates at 2000 cells per well and incubated for 1-5 days. MTT solution (0.5 mg/mL) was then added at 37 ℃ for 4 h and the media was replaced by 150 μl DMSO. Absorbance at 570 nm was then assessed by microplate reader. There were 5 replicates per group, and the experiment was conducted independently three times.

Plate clone formation assay

Stable transfected cells were seeded in 6-well plates at a density of 1000 cells per well, then cultured for 8 days. Cells were washed with PBS, fixed with 4% polyformaldehyde for 20 min, and followed by 1% Crystal Violet Staining Solution for 20 min after discarding solution. Finally, the number of colonies in each well were counted under the microscope.

Invasion assay

Matrigel (BD Biosciences) was used to coat the surface of Transwell migration chambers (Corning, Shanghai, China). Next, 5×10^4 cells were added into the upper chamber in a final volume of 100μl. In the lower chamber, 30% FBS media was added to serve as a chemoattractant. After incubated with 24 h, cells on the membrane of the lower chamber were fixed using 4% paraformaldehyde, stained using 0.1% crystal violet, and then a total of five random microscopic fields were counted. This assay was conducted in triplicate.

RT-PCR and qRT-PCR

Trizol (Invitrogen, USA) was used for all RNA isolation. Reverse transcriptions were performed by PrimeScriptTM RT reagent Kit (Takara, China) based on provided directions. RT-PCR reaction procedures were as follows: 37 ℃ for 15 min; 85 ℃ for 5 s; 4 ℃ for 10 min. SYBR®Premix Ex TaqTM (TaKaRa) was used for qRT-PCR based on provided directions with a StepOne-Plus System (Applied Biosystems). β-actin and GAPDH were served as loading control. Each sample was done in triplicate. Primer sequences are given in Table S4.

Transfection

siRNAs targeting MTA2TR, MTA2, ATF3, HIF-1α, as well as appropriate negative controls came from RiboBio (Guangzhou, China). MTA2TR overexpression vector, ATF3 overexpression vector and empty vector came from GeneChem (Shanghai, China). MTA2 overexpression vector and empty vector came from Vigene Biosciences (Shandong, China). For stable transfection, MTA2TR siRNA was constructed into lentiviral vector, with empty vector as a negative control. Lipofectamine 2000 (Invitrogen, USA) was used for cell transfection.
